# Capture, Movement, Trade, and Consumption of Mammals in Madagascar

**DOI:** 10.1371/journal.pone.0150305

**Published:** 2016-02-29

**Authors:** Kim E. Reuter, Haley Randell, Abigail R. Wills, Totozafy Eric Janvier, Tertius Rodriguez Belalahy, Brent J. Sewall

**Affiliations:** 1 Temple University, Department of Biology, Philadelphia, Pennsylvania, United States of America; 2 Mpingo Conservation & Development Initiative, Kilwa Masoko, Tanzania; 3 Département des Sciences de la Nature et de l’Environnement, Université d’Antsiranana, Antsiranana, Madagascar; Northern Illinois University, UNITED STATES

## Abstract

Wild meat trade constitutes a threat to many animal species. Understanding the commodity chain of wild animals (hunting, transportation, trade, consumption) can help target conservation initiatives. Wild meat commodity chain research has focused on the formal trade and less on informal enterprises, although informal enterprises contribute to a large portion of the wild meat trade in sub-Saharan Africa. We aimed to provide a more comprehensive understanding of the formal and informal components of these commodity chains by focusing on the mammalian wild meat trade in Madagascar. Our objectives were to: (1) identify hunting strategies used to capture different wild mammals; (2) analyze patterns of movement of wild meat from the capture location to the final consumer; (3) examine wild meat prices, volumes, and venues of sale; and (4) estimate the volume of wild meat consumption. Data were collected in May-August 2013 using semi-structured interviews with consumers (n = 1343 households, 21 towns), meat-sellers (n = 520 restaurants, open-air markets stalls, and supermarkets, 9 towns), and drivers of inter-city transit vehicles (n = 61, 5 towns). We found that: (1) a wide range of hunting methods were used, though prevalence of use differed by animal group; (2) wild meat was transported distances of up to 166 km to consumers, though some animal groups were hunted locally (<10 km) in rural areas; (3) most wild meat was procured from free sources (hunting, gifts), though urban respondents who consumed bats and wild pigs were more likely to purchase those meats; and (4) wild meat was consumed at lower rates than domestic meat, though urban respondents consumed wild meat twice as much per year compared to rural respondents. Apart from the hunting stage, the consumption and trade of wild meat in Madagascar is also likely more formalized than previously thought.

## Introduction

Consumption of wild meat is important for nutritional [1,2] and cultural reasons [3]. However, it also constitutes a threat to many animal species [4] especially wild mammals [5]. In the Congo Basin alone, up to 4.5 million tonnes of wild meat are consumed per year [6] and hunting occurs at a rate six times higher than is sustainable [7]. Growing urban populations and increasing consumer affluence has increased demand for wild meat across sub-Saharan Africa [8]. This has depleted wild animal populations and increased wild meat prices [8]. Increased sale prices and market demand encourage hunters to switch to more efficient hunting methods [3]. In addition, as habitats near large urban markets become depleted of wildlife [8], hunters partner with a series of formal and informal actors to transport and sell wild meat to consumers at distances far removed from the hunting location [7,9]. An understanding of how wild animals are hunted, and how wild meat is moved, traded, and consumed using formal and informal enterprises—referred to as the commodity chain for wild meat—is needed to safeguard species from unsustainable hunting [4,10].

Past commodity-chain research suggests that the links between hunters and consumers are often well developed [4,5,10], though the length of the chain—the number of actors who move the wild animal from its natural habitat to the consumer—can differ [5]. In general, there are four principal stages in the process of moving wild meat through the commodity chain: (1) the hunting of wild animals or their live capture (followed by killing at a later stage); (2) the transport of the animal (body parts or the whole animal either dead or alive) away from the capture location to the point of exchange or consumption [3,4,10]; (3) exchange of wild meat or the live animal with transporters, other traders, or consumers via sale [1,3,4], via barter for non-monetary goods or to acquire social favors, or via gifting to relatives or friends; and (4) consumption for nutritional [1,2] or cultural reasons [3]. Consumption is the most-studied stage of the commodity chain and has been examined across Africa [11,12], Asia [13], and South America [6]; the other three stages are less well understood.

Past research on wild meat commodity chains has focused on Central and West Africa, where the trade is relatively formalized (involving a consistent set of actors and established venues). Accordingly, most past research has focused on the formal trade [5] rather than examining informal enterprises (small businesses that lack large capital investments, that may be ephemeral, or that exist outside government oversight) [14]. However, informal sectors account for over 50% of the GDP and employment in many African countries [14]. Moreover, most wild meat is illegally extracted [3] and is traded via informal enterprises [14]. Similarly, the emphasis of past studies has been on measuring the flow of goods that have market value [5], and have focused less on barter, gifts, and non-market exchanges of wild meat. Thus, excluding informal and non-market exchanges may lead to an incomplete understanding of the commodity chain in areas such as Madagascar, that have a less organized wild meat trade, where there are often household-to-household sales [15,16], where most wildlife biomass (66%) in rural areas is hunted illegally [16], and where people are known to barter wild meat for other items. A study of Madagascar could provide a more comprehensive understanding of commodity chains for wild meat, including informal and non-market exchange. Such study could also prove beneficial for conservation because hunting of Madagascar’s highly endemic mammal taxa may be unsustainable for lemurs [15], fosa [15], and bats [17], and because political instability following a 2009 coup d’état may have further increased trading volume [18].

Past research has clarified some basic elements of Madagascar’s wild meat commodity chain. A diversity of taxa are hunted (including protected species), hunting occurs throughout the year including outside established seasons in game animals, and a wide variety of hunting and capture methods are employed [19,20,21]. The sparse data available from Madagascar [19,22] suggest wild meat is transported only short distances from hunting locations (4.40 ± 2.90 km [15]; 15 km [23]), perhaps because—unlike in other sub-Saharan countries [4]–wild meat in Madagascar is usually sold fresh and not smoked. Wild meat is sold for < 1–7 USD/kg in rural areas of the country [16,24], with preferred wild meat costing more, and less preferred wild meats costing less than, domestic meat [21]. Consumption of wild meat is consumed occasionally; most households (95%)[15] and most people (60%)[25] consume wild meat at least once a year though it appears in just 1.3% of meals [26]. It is not clear how the commodity chain varies by animal taxa or between rural and urban areas in Madagascar.

**Fig 1:**
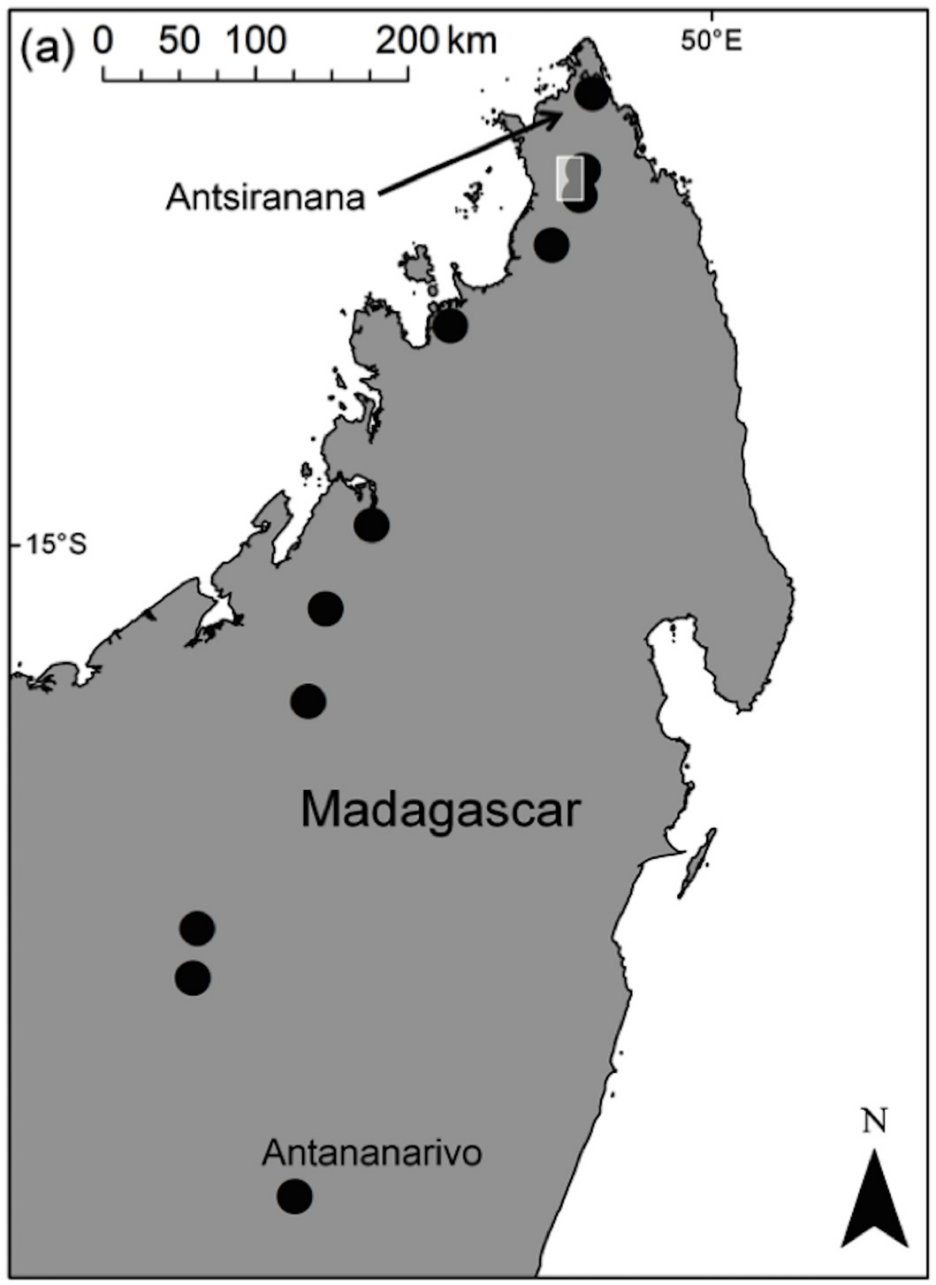
Urban towns in central and northern Madagascar where interviews were conducted. The shaded rectangle shows the location of rural towns (in the vicinity of the Ankarana National Park) where interviews were conducted but exact locations are not shown to protect respondent anonymity. Figure originally published in Reuter et al. 2015 [27].

Our aim in this study was to improve understanding of the formal and informal commodity chain for wild meat in Madagascar (Fig 1). Our objectives were to: (1) identify the principal methods used to hunt wild animals of different groups and the timing of hunting; (2) analyze the patterns of movement of different types of wild meat from the capture location to the final consumer; (3) examine the prices, volumes, and venues of sale of wild meats; and (4) estimate the volume of wild meat consumed. For objectives 2–4, we used the movement, sale, and consumption of domestic meat—i.e., meat from animals like zebu (a subspecies of cattle found in Madagascar), chickens, and pigs that were raised by farmers or ranchers—as a point of reference for comparison with wild meat.

For our first objective, we determined hunting methods and timing. We hypothesized that hunting methods would: (1A) differ between urban and rural respondents due to the different resources available to hunters in each setting; (1B) differ before and after 2009, due to reports of decreased enforcement on hunting following a political coup d’état [18]; (1C) focus on slingshots, spears, and other less-efficient tools when wild meat was for personal consumption; (1D) focus on nets, firearms, and other highly-efficient tools when wild meat was for sale. In addition, we hypothesized that the timing of hunting would: (1E) be year-round for all animals except for tenrecs, which estivate for a portion of the year [24]; and (1F) that rural respondents would be more likely than urban ones to report year-round hunting, given the potential of rural respondents for living in closer proximity to remnant animal habitats. For the same reason, we also hypothesized that (1G) the proportion of consumers who directly hunted wild meat would be lower in larger towns.

**Fig 2:**
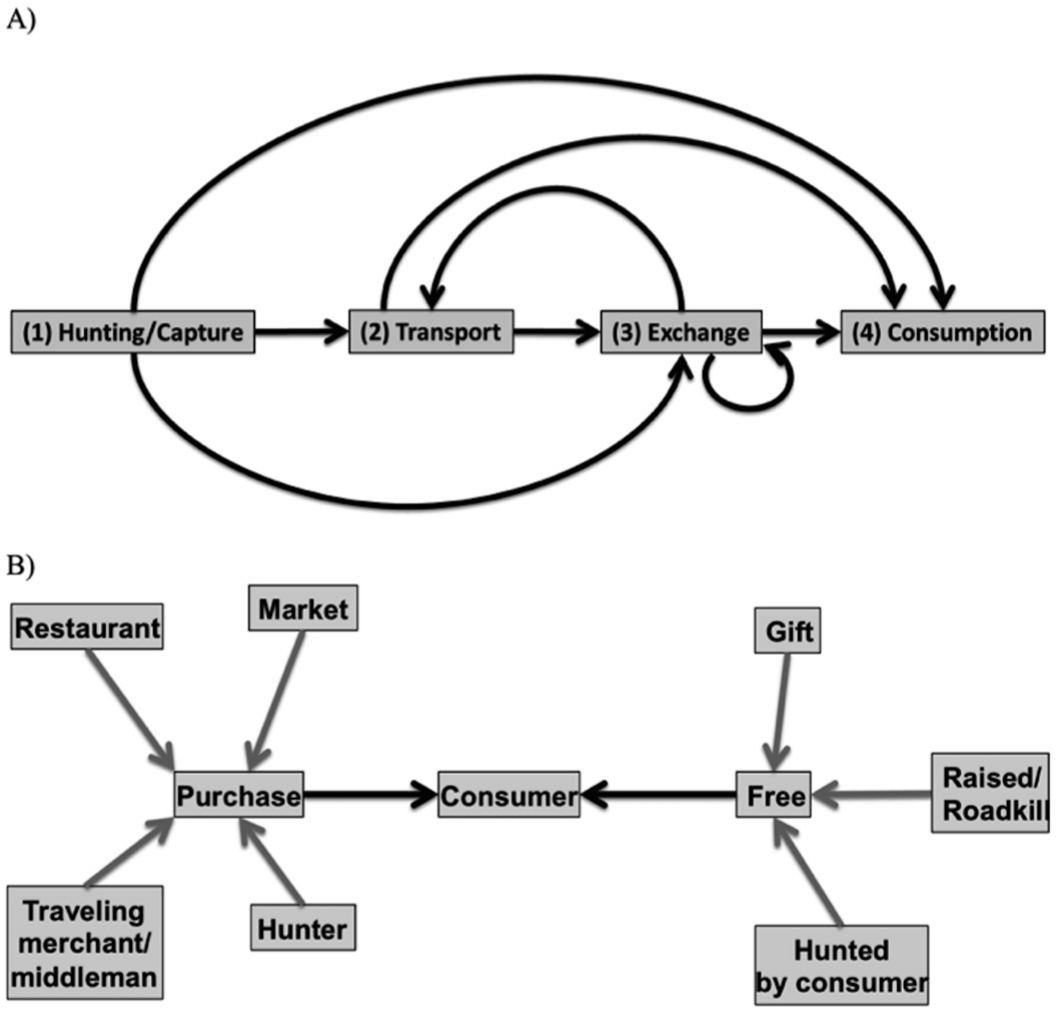
Conceptual commodity chain models (A) and the sources from which consumers procure wild meat (B). The commodity chain (A) is not always linear and does not always include all actors; for example, in some cases, the consumer is also the hunter. In image (B) there are two tiers of arrows; the arrows that connect peripheral boxes to the “purchase” and “free” boxes represent direct quantities measured (black arrows) while the arrows connecting the “purchase” and “free” boxes to the consumer represent sums of the peripheral boxes of each type (gray arrows).

Our second objective was to describe the patterns of movement of wild meat from its capture location to the final consumer, including differences by location and animal group (Conceptualized in Fig 2). Given the short transport distances found in past studies [15,23], we hypothesized (2A) that wild meat would generally be sourced locally. We also hypothesized that (2B) some animal groups that are attracted to human-dominated landscapes would be hunted in close proximity to towns (e.g., wild cats [15]), whereas (2C) other groups of animals (e.g., lemurs) that avoid human-dominated landscapes would be hunted farther away. However, because less forest habitat for wild animals may be present near large towns, we hypothesized that (2D) the transport distance would increase with a town’s population. In addition, because domestic meat is often raised near human settlements and habitats of wild animals may be remote from towns, we hypothesized that (2E) wild meat would be sourced at greater distances than domestic meat. Finally, given that most wild meat is caught illegally in Madagascar [16], we hypothesized that to avoid drawing attention to illegal wild meat, (2F) the volume of wild meat transported on the inter-city transit system would be low.

Our third objective was to examine the prices, volumes, and venues of sale of wild meats, relative to domestic meat. We hypothesized that due to increased distances and costs of transport of wild meat in towns with higher populations [8] and increased costs of living in larger towns, (3A) prices would be positively correlated with the population of a town. Based on the urban meat price estimates [21] we hypothesized that (3B) average prices of wild and domestic meat would be similar, but that wild meat prices would vary by animal group (bat meat would be cheaper than domestic meat; wild pig meat would be the same price as domestic meat; tenrec meat would be more expensive than domestic meat). In addition, we hypothesized (3C) that the cost of wild meat would differ depending on the source from which it was purchased. Specifically, due to profit-taking by middlemen, we hypothesized that (3D) the per-unit price paid by the consumer would increase when wild meat was not purchased directly from the hunter. In terms of the volume of sale, given that most wild meat is caught illegally in Madagascar [16], we hypothesized that to avoid drawing attention to a potentially illegal activity, wild meat would be (3E) sold less at markets than domestic meat and (3F) traded less frequently through established venues than domestic meat. In addition, because wild meat hunting may require specialized methods, tools, or knowledge, or access to remnant forest habitats, and because of past research suggesting substantial portions of wild meat are sold to markets and restaurants [21], we hypothesized that (3G) most consumers would obtain wild meat from a third party rather than hunting it themselves. Finally, because impacted habitats near larger towns might increase the logistics and distance associated with obtaining wild meat by urban consumers, we hypothesized (3H) that the proportion of wild meat purchased from a middleman (rather than hunted by the consumer or purchased directly from the hunter) would increase with the population of a town.

Our fourth objective was to estimate the relative volume of consumption of wild meat. Given that price [16] and preference [21] differ by animal and impact the likelihood of consumption, we hypothesized that (4A) the volume of consumption would differ by animal group. In addition, given that regular consumption of wild meat is low in Madagascar [21,26] we hypothesized that (4B) the volume of consumption would be lower in wild meat that in domestic meat for all animal groups.

## Methods

### Study site

Data were collected (May-August 2013) at twelve urban and nine rural towns in central and northern Madagascar (Table 1). Urban towns were located along the 1,092 km-long highway connecting the northern regional capital Antsiranana with the national capital Antananarivo, located in the center of the country (Fig 1). Rural towns were located around the perimeter of Ankarana National Park, a large protected area (18220 ha) next to this highway that contains one of the world’s highest densities of primates [28].

**Table 1:** Table of study sites included in research project.

Town	Number of interviews					Population	Distance from Antananarivo
	MC	R	M	SM	TI		
Antananarivo	199	150^1^	31	3	18	1,054,649*	0 km
Ankazobe	63	-	26	-	-	13,085*	92 km
Mahatsinjo	58	-	4	-	-	15,000*	177 km
Andriba	122	-	12	-	-	32,000*	198 km
Antsiafabositra	70	-	5	-	-	8,328*	243 km
Tsararivotra	32	-	5	-	-		496 km
Andrevorevo	40	-			-		582 km
Antsohihy	60	2	17	-	1	105,317*	668 km
Ambanja	55	2	23	-	8	28,468*	865 km
Ambilobe	99	-	23	-	4	56,427*	962 km
Ankarana National Park							
*Ambondromifehy*	30		N/A			5000	
*Ampasinbengy*	30		N/A			1997	
*Andranokoho*	33		N/A			2000	
*Lambondry*	34		N/A			120	
*Mahamasina*	28		N/A			650	
*Marotaolana*	30		N/A			175	
*Marovato*	30		N/A			400	
*Matzaborimanga*	30		N/A			400	
*Tsarakibany*	30		N/A			250	
Aniverano Nord	90	-	15	-	-	15,000*	1025 km
Antsiranana	180	9	193	-	30	87,569*	1100 km
**Total:**	**1343**	**163**	**354**	**3**	**61**		

The number of interviews completed at each study site are listed by interview type. The distance of population centers from Antananarivo are listed; Antananarivo was the southernmost population center included in this study while Antsiranana was the northernmost. Population estimates flagged with (*) are estimates retrieved from the Ilo database [29]. All other estimates were provided by locally elected officials. MC: meat consumption interview; R: restaurants; M: open-air markets; SM: supermarkets; TI: transportation interviews.

^**1**^150 restaurants were approached (105 in person and 45 by phone) for information regarding their history of wild meat; 140 restaurants had never sold wild meat and provided no additional information and 10 restaurants were given a full meat-seller interview.

### Legality of wild meat hunting and consumption in Madagascar

Hunting in Madagascar is regulated using legal instruments, with the right to hunt belonging to the state and allowed through a permit-based hunting system [30]. Regulations restrict the species that can be hunted, hunting methods, and hunting seasons; they also prohibit the possession, transport, sale, and export of illegally acquired animals (S1 Appendix).

### Research permissions

Research design was approved by an ethical review committee for human subjects research (Temple University Institutional Review Board), and field research was conducted under the authorization of the Madagascar Ministry of Water and Forests and Madagascar National Parks. Permission to conduct research was also gained in each town from the highest ranking, locally elected official. Verbal informed consent was secured from all research participants prior to the onset of data collection; the Institutional Review Board approved the use of verbal consent. Written consent was not obtained due to concerns with literacy levels in the respondent pool. Verbal consent was documented during the administration of the interview; interviews were not administered without verbal consent.

### Social surveys

Data were collected from the following actors of the commodity chain: (1) hunters (informal enterprises); (2) inter-city transporters (formal enterprises); (3) meat-sellers (both formal and informal enterprises; open-air markets, restaurants, and supermarkets); (4) and consumers. No hunters reported obtaining a permit or regularly selling wild meat; thus all of the hunters interviewed were considered part of the informal trade. In this study, a ‘restaurant’ was any business that sold prepared foods for consumption, including Malagasy-owned restaurants targeting Malagasy customers referred to as *gargottes* or *hotely* and the more expensive Malagasy and expatriate-owned restaurants targeting wealthier Malagasy individuals and foreigners. Meat-sellers involved in formal enterprises included the more expensive restaurants, some of the larger *hotely*, the supermarkets, and the permanent open-air market stands, whereas meat-sellers involved in informal enterprises included the smaller *hotely* and the intermittent open-air market stands (e.g., those only open on some market days).

Data were collected using three semi-structured interviews [31] with three protocols and respondent groups: (1) meat consumption interviews with members of the community; (2) meat-seller interviews targeted to employees at meat selling venues; and (3) transportation interviews targeted to employees of inter-city transportation companies (bus and boat). Data from hunters were collected through the meat consumption interviews; all interviewees were asked how they sourced their wild meats (including hunting) and whether they ever sold wild meat (Questions 1, 1a, and 1c in Meat Consumption Interviews, S2 Appendix). All surveys were administered by research teams composed of two individuals, including one lead researcher (KER, AW, HG) and one translator (TEJ, TRB). Surveys were administered orally in the interviewees’ language of choice (local Malagasy dialect or French). Face-to-face recruitment was used to enroll respondents into the study. Verbal informed consent was always received prior to the onset of data collection. To protect respondent anonymity, no identifying information was collected.

Meat consumption interviews were administered at select households in each town. In rural towns (range: 120–5,000 inhabitants) every 5^th^ household was sampled. In urban towns (range: 6,622–1,054,649 inhabitants) random sampling was stratified by administrative unit. To ensure independence of samples, interviews were requested with one person per household. Respondents included both males and females, were 18 years or older, and considered themselves to have a major buying power for household goods. If an eligible individual refused or if no one was present, an adjacent household was approached. Meat consumption interviews lasted an average of 11 ± 0.27 minutes (± 95% CI), and 1343 people were interviewed (Table 1); we had no prior knowledge of respondents’ histories of hunting and consuming wild meat.

The meat consumption interviews were designed to collect data on meat consumption habits (Questionnaire in S2 Appendix). In the first section, respondents were asked to discuss any type of meat consumption and provide information (including data on hunting, purchasing and other attainment methods) about recent (within the last three days) meat consumption and preferred meat types. In the second section, respondents were asked questions regarding their history of wild meat consumption (amount, species, preference), sources of wild meat (e.g. hunting or purchase), and the circumstances of consumption. In the third section, respondents were asked about any changes in their wild and domestic meat consumption as well as meat-related taboos.

Meat-seller interviews were conducted in all (n = 11) urban towns with permanent food markets or restaurants (Table 1), and were designed to collect data on the volumes and types of meat being sold in open-air markets, supermarkets, and restaurants (Questionnaire in S2 Appendix). In all but the two largest towns (Antsiranana and Antananarivo, see below), we first interviewed all meat-sellers who were available during our visit (~60–100% per town) in open-air markets at permanent stands (where meat was sold on most days) and at intermittent stands (where meat was typically sold only on weekly market days). Interviews were conducted with individuals in open-air markets without prior knowledge of their history of selling wild meat and regardless of whether they appeared to be selling wild meat at the time of the interview. In Antsiranana and Antananarivo, the large size of the towns required a modification of the sampling strategy. In Antsiranana, data were collected from randomly sampled meat-sellers in every permanent open-air market in the city. In the largest town of Antananarivo, a few meat-sellers (n = 1–5) were sampled randomly from major open-air markets in 14 quarters, spread evenly across the city. These quarters represented a wide range of communities, including areas of different ethnic, religious, and socioeconomic characteristics. We also opportunistically sampled three supermarkets (enclosed, upscale food stores with diverse merchandise) in Antananarivo.

In all towns except Antananarivo, knowledge derived from meat-sellers at open-air markets and meat consumption interviews was then used to identify restaurants with a prior history of selling wild meat. In Antananarivo, the size of the city and number of restaurants precluded the use of interview responses as the sole source of information about restaurants selling wild meat. We therefore visited 105 restaurants across 10 quarters and made telephone contact with an additional 45 restaurants spread across the city. After obtaining informed consent, we asked respondents from these Antananarivo restaurants if wild meat had ever been sold at the restaurant. If the respondent indicated the restaurant had never sold wild meat (n = 140), we ended the interview. For all others (n = 10), we completed the standard meat-seller interview.

For all types of meat-sellers in all towns, we ensured independence of samples by selecting only one worker per establishment for an interview; this was the proprietor or manager or their designee. Full meat-seller interviews (excluding initial contacts with restaurants in Antananarivo that did not result in a full meat-seller interview) lasted an average of 8 ± 0.26 minutes and we interviewed representatives from a total of 354 open-air markets, 3 supermarkets, and 163 restaurants (Table 1). Questions included: recent meat sales (species, volume, rate, and price of sale during the previous three days); lifetime sale of wild meat; methods for attaining meat (hunting, purchasing, or other methods; meat-related food taboos; and changes in type or rate of meat sales over time (Questionnaire in S2 Appendix).

Finally, transportation interviews were conducted in a few (n = 5) of the larger towns, to understand how domestic and wild meats were moved between towns. Interviews were conducted with boat and bush taxi drivers at major bus stations and ports (Table 1). Transportation interviews lasted an average of 8 ± 0.01 minutes, and 61 people were interviewed (Table 1). Respondents were asked to quantify the amount of meat transported within the last three days (including information about departure and destination points, as well as any fees associated with transportation) and their lifetime history of wild meat transportation (Questionnaire in S2 Appendix).

Pilot interviews (for all interview types) indicated that respondents typically could not identify exact species (neither from photographs nor by name) and did not always categorize animals as they might be categorized by scientists (e.g., by family and by genus). However, the general public could differentiate between broader animal groups. Therefore, and based on the feedback from the respondents in the pilot interviews, data were aggregated into the following mammal groups which are most identifiable to the Malagasy general public: lemurs (Lemuroidea), bats (Chiroptera), tenrecs (Tenrecinae), fosa (*Cryptoprocta ferox*), mongoose (Eupleridae), rats and mice (Rodentia), civets (Viverridae and Eupleridae), wild cat (*Felis silvestris*), and wild pig (*Potamochoerus larvatus*).

We acknowledge the limitations of the survey materials used in this study. First, given that the survey included questions on activities that are sometimes illegal, respondents may have under-reported sensitive information. Second, the reported lifetime rates of wild meat hunting/transport/trade/consumption should be treated as estimates, especially when respondents had interacted with a wild meat multiple times (e.g., in more than a few instances). The lifetime recall period was used as it may result in a more accurate estimate of rare events (as opposed to extrapolation of data from shorter time periods) and because it allowed for an analysis of trends over time (as reported in Reuter et al. in press [32]); a study on wildlife consumption in Madagascar found that recalls of the prior year were more accurate than extrapolation of recalls of the prior month [33]. Third, the time frame of the study was limited (surveys conducted over a four-month time period; some questions used a 3-day recall period). However, many survey questions were phrased in a way to collect data about respondents’ interactions with wild meat throughout the year (see S2 Appendix). It should be noted that questions generally utilized a 3-day diet recall when it was likely that respondents would not be able to accurately provide data for a longer time period (e.g., respondents would not be able to state how many times in their lifetime they had consumed chicken; diet recall in Madagascar for non-rare food-related events decreases substantially after three days [26]). The geographic scope of the study prohibited data collection over multiple time points. In contrast, much of the data presented here is based on questions that asked respondents to describe their lifetime consumption patterns. It should be noted that we conducted interviews during the time of year when food is relatively abundant. Therefore, under the assumption that wild meat is consumed more in times of food scarcity, and if our interview period did affect respondents’ responses, then we would assume that the data presented in this study are conservative estimates.

### Analysis

Results are presented as means with 95% confidence intervals and towns are replicates unless otherwise noted (when n < 20). All analyses were completed using JMP statistical software (JMP, Version 10. SAS Institute, Cary, NC, 1989–2007).

For objective one, Pearson’s Chi-squared Tests were used when n > 1000 and Fisher’s Exact Tests were used when n < 1000 in analyses of hunting methods (hypotheses 1A-1D). A Bonferroni correction was used when determining significance in multiple post-hoc tests to maintain experiment-wise error at an alpha level of 0.05. Data were eliminated from analyses in 16 cases where respondents were not clear whether they used a slingshot or a firearm.

For hypothesis (1E), respondents were not asked directly whether they hunted within a specific season, but many volunteered an explicit time. We used these data to analyze seasonality of hunting by quarter of the year in which most hunting took place (Quarter 1: January—March; Quarter 2: April—June; Quarter 3: July—September; Quarter 4: October—December). In most towns, Quarters 1 and 4 are warmest and receive the most rain, while Quarter 3 is the coolest and has the least rain, and Quarter 2 is intermediate. For hypothesis (1G), we regressed the natural log of town population against the arcsine transformed percentage of respondents consuming wild meat; towns were replicates.

For objective two, when the source location of a meat was a known town or landmark located on a road, the distance between that location and a respondent’s town of residence was calculated from satellite images and maps using the shortest route along the country’s road network. When the source location was not on a road, the distance was the sum of the shortest route along roads to the closest point, and the direct overland distance from that point to the source location (without considering walking trails, which could not be detected in the satellite images and maps). When a single respondent listed more than one source location, distances were averaged for that respondent. For hypotheses (2D) and (2E), a mixed effects model was used with the natural log of ‘town population’, ‘type of meat’ (i.e. wild or domestic), and ‘animal group’ (nested within ‘type of meat’) as the fixed effects, ‘town’ as the random effect, and the natural logarithm of distance to purchase location (in km) as the response. For wild meat, we also separately examined a model with the natural log of ‘town population’ and ‘animal group’ as the fixed effects, ‘town’ as the random effect, and the natural logarithm of distance to hunting location (in km) as the response.

For objective three, prices are in Malagasy Ariary (2,197 Ariary = 1 USD)[34]. Because few meat-sellers in our sample sold wild meat (see results for hypothesis 3E below), we used responses from meat consumption interviews to estimate wild meat prices (S4 Table). Only price data provided by respondents referring to recent purchases (during 2012 and 2013) were used to decrease inaccuracies from incorrect information recall over longer time periods. For hypothesis (3B) a mixed effects model was used with ‘type of meat’ (i.e. wild or domestic) and ‘animal group’ (nested within ‘type of meat’) as fixed effects, ‘town’ and ‘unit of purchase’ (i.e. whether the purchase was of a whole animal, kilogram of meat, or plate of meat at a restaurant) as random effects, and the natural log of the price of meat (Malagasy Ariary) as the response. For hypothesis (3C) a mixed effects model was used with ‘purchase venue’ (i.e. hunter, open-air market, restaurant, or middleman) as the fixed effect, ‘town’, ‘animal group’, and ‘unit of purchase’ as random effects, and the natural log of the price of meat (Malagasy Ariary) as the response. For hypothesis (3H), we regressed the natural log of town population against the arcsine transformed percentage of consumers buying wild meat from middlemen; towns were replicates.

For objective four, hypotheses (4A) and (4B) used a mixed effects model with ‘type of meat’ (i.e. wild or domestic meat), and ‘animal group’ nested within ‘type of meat’ as the fixed effects, ‘town’ as the random effect, and the natural log of consumption frequency (number of times consumed per year) as the response.

## Results

### Capture/hunting of wild animals (objective one)

Respondents to the meat consumption interviews (n = 1343) across all towns reported 1463 records of hunting animals, of which 84% provided information about the hunting method(s) used. Many urban and rural respondents (54 ± 10% and 46 ± 23%, towns as replicates) had hunted a wild animal at least once in their lifetimes.

**Fig 3:**
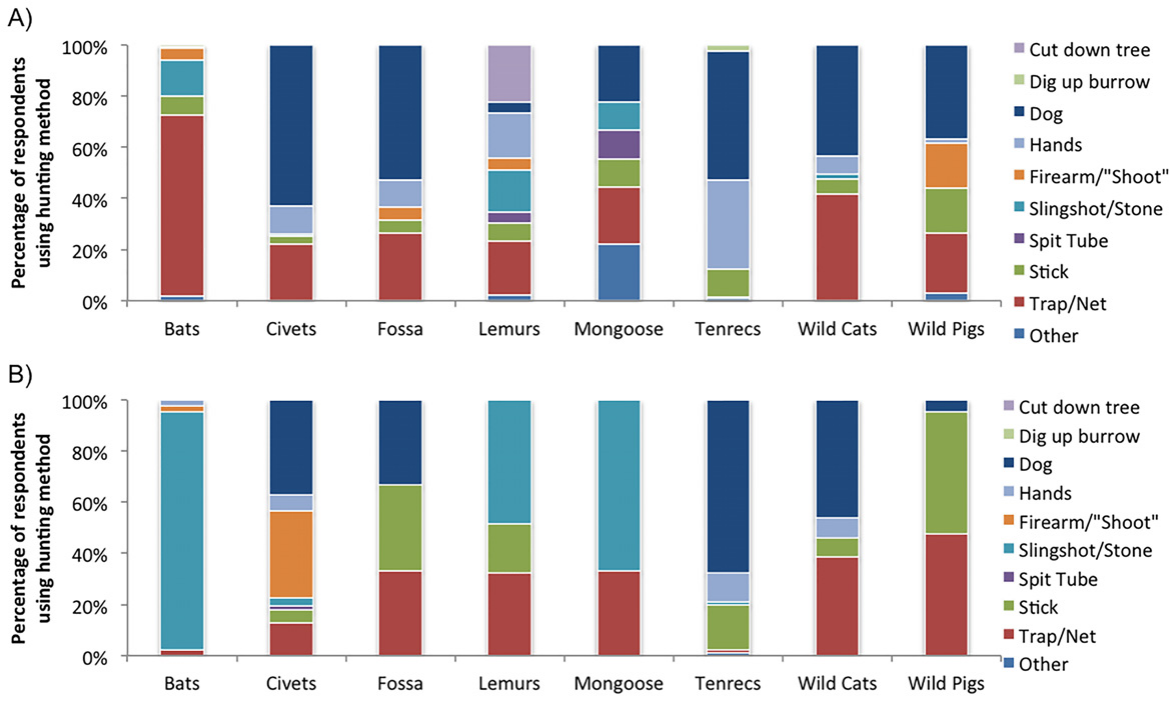
Hunting methods used to capture wild mammals by urban (1A) and rural (1B) respondents (percentages). Individuals are replicates; data taken from meat consumption interviews. Hands = catching with hands by one or multiple people. Trap/Net = includes nets, cable traps, bait with traps. Spit Tube = a blow tube or a device to shoot darts. Stick = includes hitting animal with stick or spearing animal with stick. Individuals are replicates. Other = includes other hunting methods (e.g. fire, using a cross bow).

The most popular hunting method included tracking and capturing the animal with a dog (33%, individuals are replicates), followed by: catching with a trap/net (21%); catching with bare hands, alone or with other people (17%); striking with a rock hurled from a slingshot or thrown (11%); hitting with a stick or stabbing with a spear (10%); shooting with a firearm (3%); felling the tree in which the animal was hiding (2%); digging up the animal’s burrow (1%); and darting with a blow tube or spit tube (< 1%). Hunting methods differed significantly by animal group (Pearson’s Chi-Squared Test, χ^2^ = 1257.331, p < 0.0001; Fig 3). Lemurs were the only animals hunted by cutting down the entire tree; this hunting strategy was often associated with hollow-dwelling nocturnal lemurs. Tenrecs were the only animals hunted by digging up their burrows.

In accordance with hypothesis (1A), hunting methods differed between urban and rural respondents (Pearson’s Chi-Squared Test, χ^2^ = 215.107, p < 0.0001; Fig 3). Some methods/tools were reported exclusively by respondents living in urban areas: blow tubes (n = 8), firearms (n = 32), cutting down trees (n = 29), and digging up burrows (n = 14). Some animal groups were hunted using different methods when urban respondents hunted them, as opposed to rural respondents, including: 1) bats (post-hoc Fisher’s Exact Test, p < 0.0001); 2) lemurs (p < 0.0001); 3) tenrecs (p < 0.0001); 4) and wild pigs (p < 0.0001). Sample sizes were too small to test fosa and mongoose, and there was no difference in how wild cats or civets were hunted.

**Fig 4:**
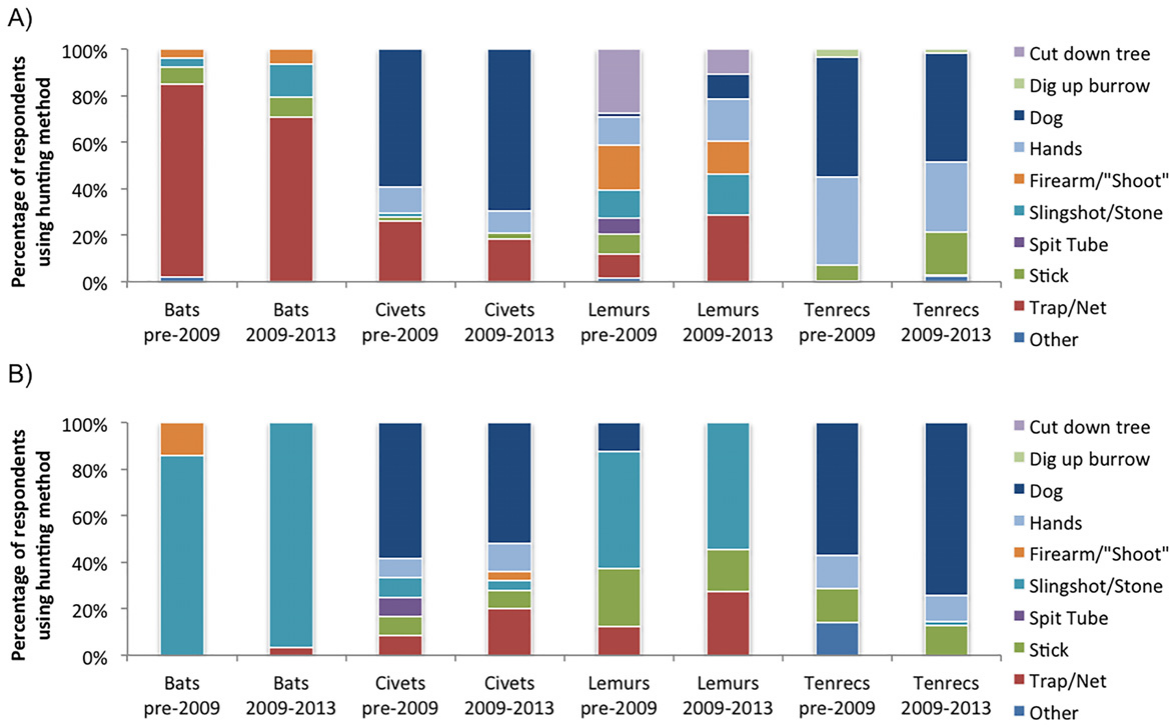
Hunting methods used pre-2009 and between 2009–2013 for (A) urban- and (B) rural hunters (percentages). Data taken from meat consumption interviews. Only animal groups with ≥5 respondents in each time class are presented. Individuals are replicates. Hands = catching with hands by one or multiple people. Trap/Net = includes nets, cable traps, bait with traps. Spit Tube = a blow tube or a device to shoot darts. Stick = includes hitting animal with stick or spearing animal with stick. Individuals are replicates. Other = includes other hunting methods (e.g. fire, using a cross bow).

There was mixed support for hypothesis (1B). When all hunting reports were aggregated, the type of hunting method differed in the pre-2009 period and the 2009 to mid-2013 period (Pearson’s Chi-Squared Test, χ^2^ = 64.455, p < 0.0001). However, there was no significant shift between periods in hunting strategies except among urban respondents who hunted tenrecs (post-hoc Fisher’s Exact Test, p = 0.0006; Fig 4).

**Fig 5:**
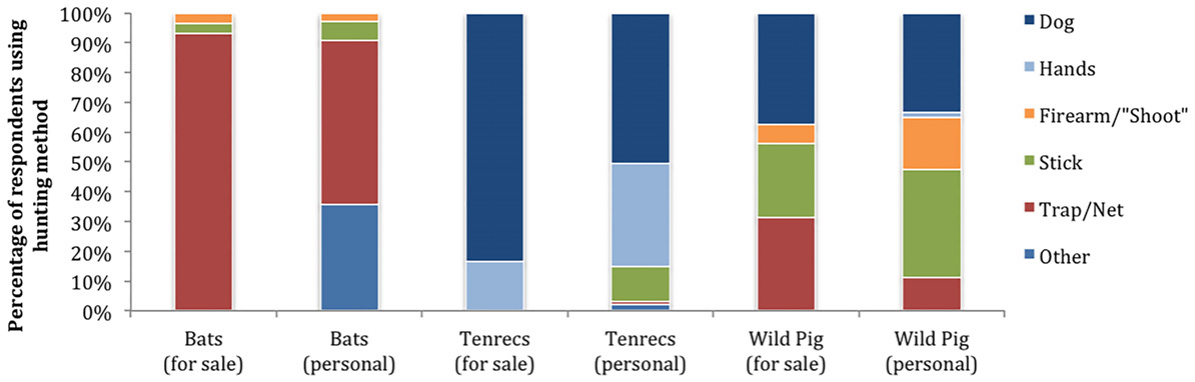
Comparison of hunting methods used to capture wild mammals for sale and for personal consumption (percentages). Individuals are replicates; data taken from meat consumption interviews. Hands = catching with hands by one or multiple people. Trap/Net = includes nets, cable traps, bait with traps. Spit Tube = a blow tube or a device to shoot darts. Stick = includes hitting animal with stick or spearing animal with stick. Individuals are replicates. Other = includes all other hunting methods (e.g. slingshots, fire, using a cross bow).

Forty-three individuals across ten urban and four rural towns had caught wild meat for commercial purposes themselves (8 ± 3% of people in towns where at least one individual had caught wild meat for sale). Most individuals had only caught and sold one type of wild meat, though five (12% of n = 43) had caught and sold two different types of wild meat, thereby providing 48 records of wild meat hunting for commercial reasons. In accordance with hypotheses (1C, 1D) there was a difference between the hunting methods used when an animal was caught for personal consumption compared to sale (Fisher’s Exact Test, p < 0.0001; Fig 5). Though sample sizes were too small to examine statistically the differences in hunting methods within individual animal groups, some animals captured for resale tended to be caught using more efficient methods than animals captured for personal consumption. In particular, bats caught for personal consumption were caught using nets proportionately less than bats caught for resale (Fig 5), and tenrecs captured for personal consumption were caught using a variety of methods while tenrecs caught for sale were almost always captured using dogs (Fig 5).

**Fig 6:**
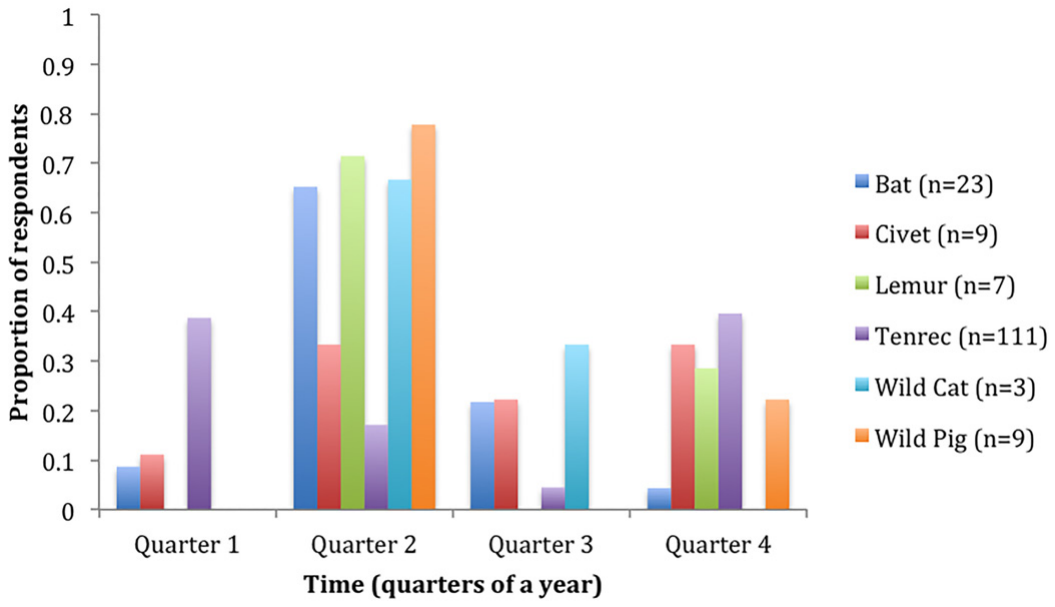
Time of year when respondents hunt for different types of wild mammals (individuals are replicates). Sample sizes are listed for each animal group. Data taken from meat consumption interviews. Quarter 1: January-March; Quarter 2: April-June; Quarter 3: July-September; Quarter 4: October-December.

Of the 1463 hunting records, 259 (17.7%) referenced hunting during a specific time of the year (e.g. an informal or formal hunting season) for at least one animal group. In accordance with hypothesis (1E), tenrecs were the only group where over 10% of both urban and rural respondents (towns as replicates) mentioned a hunting season (S1 Fig), though in general for most animal groups, respondents reported hunting animals in the second (April-June) and fourth quarter (October-December) more than other times of the year (Fig 6). In contrast, although we did not ask meat-sellers whether hunting seasons impacted the sale of meat, a few respondents (at an open-air market in Antsiranana and at three restaurants in Antananarivo) noted that the third quarter (June-August) represented the season when wild pig meat was easier to procure and sell. Contrary to hypothesis (1F), both urban and rural respondents reported hunting seasonally at similar proportions for each animal group (all Fisher’s Exact Tests, p > 0.14; wild cat, mongoose, and fosa not tested due to small sample sizes). Finally, contrary to hypothesis (1G), the percentage of consumers who directly hunted wild meat did not change with the population of a town (Regression, ln(population) = 0.05, F_1,16_ = 1.3815, p = 0.24, R^2^ = 0.01, towns as replicates with percentage of consumers arcsine transformed).

### Movement of wild and domestic meat (objective two)

**Fig 7:**
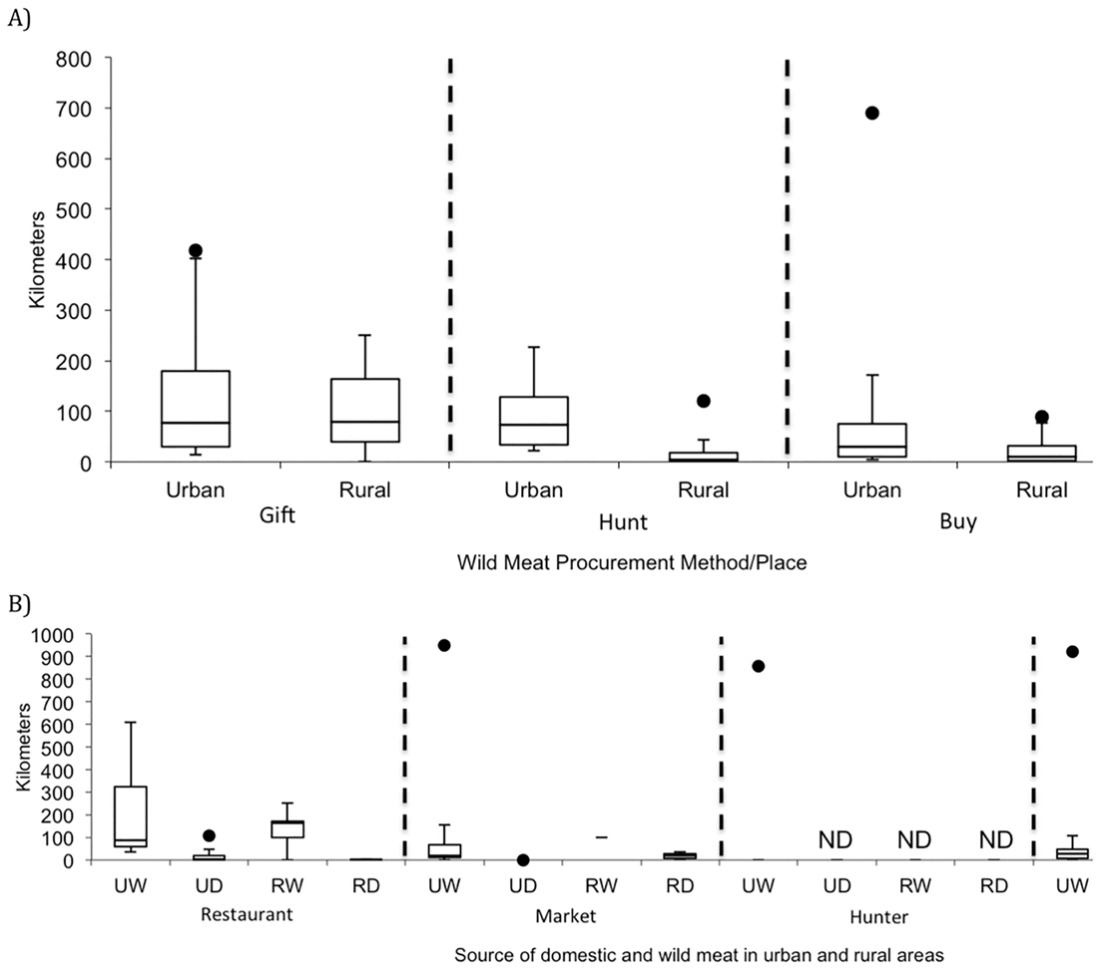
Distances that meat was transported prior to consumption. Data taken from meat consumption interviews. Distances that meat was transported to, or that urban and rural consumers traveled, to (A) procure wild meat as a gift, from hunting, or purchase; and (B) domestic and wild meat purchased from restaurants, markets, hunters, and middlemen. The whiskers extend to data points within 1.5 times the interquartile range; black circles indicate outliers. Towns are replicates. ND: No Data; UW: Urban seller of wild meat; UD: Urban seller of domestic meat; RW: Rural seller of wild meat; RD: Rural seller of domestic meat.

The 1343 respondents of the meat consumption interviews provided 1879 records of wild meat consumption and 1234 records of domestic meat consumption with information needed to calculate the distances traveled during the procurement of meat by consumers. Contrary to hypothesis (2A), wild meat obtained by urban respondents was often sourced from areas at an intermediate distance or far from (10–700 km) a respondent’s town of residence, whereas respondents in rural regions obtained wild meat more locally (Fig 7A; S1 Table). In contrast, data from the meat-seller interviews indicated wild meat sold in urban markets and restaurants tended to be sourced from areas close to or at an intermediate distance from those establishments (within 42 kilometers; S2 Table).

For hunting of wild meat, 42.0% of 1089 respondents from the meat consumption interviews traveled to hunt wild meat. In accordance with hypotheses 2B and 2C, respondents were always least likely to travel to hunt animal groups that are attracted to human-dominated landscapes (0% of respondents traveled beyond their home neighborhood to hunt rats; 25.7% traveled to hunt wild cats, 34.7% traveled to hunt civets), whereas the respondents were most likely to travel to hunt animal groups that avoid human-dominated areas (51.4% traveled to hunt lemurs). Of the cases where the respondent traveled to hunt wild meat, animal group was also an important predictor of distance traveled (Mixed Effects Model; ln(population) = 0.38, F_1, 18.01_ = 30.74, p < 0.0001; animal group, F_7, 413_ = 4.33, p = 0.0001; R^2^ = 0.36). Although respondents traveled farther to hunt mongooses and bats than they traveled to hunt tenrecs and wild pigs, there were no differences evident among other animal groups, including those that were attracted to or avoid human-dominated landscapes (Tukey HSD post-hoc test). In addition, in accordance with hypotheses (2D) and (2E), a greater percentage of people traveled to purchase wild meat (22.3% of 703 respondents purchasing wild meat traveled beyond their home neighborhood) than to purchase domestic meat (11.1% of 1234 respondents). Further, of those consumers who did travel, the distance traveled to purchase meat from any source (including restaurants, markets, hunters, and middlemen) was greater for wild meat than domestic meat, and the purchase distance increased more with the population of a town for wild meat than for domestic meat (Nested Mixed Effects Model; ln(town population) = 0.13, F_1, 19.3_ = 2.75, p = 0.11; type of meat = 1.17, F_1, 267.6_ = 81.0, p < 0.0001; animal group[type of meat], F_7, 263.2_ = 2.57, p = 0.014; ln(town population) by type of meat interaction = 0.29, F_1, 276_ = 122.4, p < 0.0001; R^2^ = 0.78). Wild meat sold in urban areas was consistently sourced from farther away than wild meat sold in rural areas or domestic meat sold in urban or rural areas (Fig 7B). Similarly, the distance traveled to hunt wild meat was greater in larger towns (see model presented above for 2B and 2C).

From the transportation interviews (n = 61), most transporters (65 ± 25%, towns as replicates) had transported meat in the three days prior to their interview. Few transporters interviewed (2.7 ± 5.2% or n = 4) had transported wild meat (including bats, tenrecs, and wild pigs) in the three days prior to the interview (all who had transported wild meat in the three days prior, were interviewed in Antsiranana). All transporters who had moved wild meat in the past three days had also transported domestic meat during that time period as well. One-third (34 ± 27%) of transporters had transported wild meat at some point in the past. In accordance with hypothesis (2F), the percentage of drivers who had ever transported chicken (62 ± 24%) was higher than the percentage of drivers who had ever transported any type of wild meat (means for five different wild meats ≤ 24%, S3 Table). Wild meat was transported by respondents less frequently than domestic meat. However, when wild meat was transported, the number of carcasses of individual animals transported per trip were similar to that of a shipment of domestic animals (Column: “When transporting, how many are transported per trip?”; S3 Table); in other words, shipments of wild meat and domestic meat were generally similar in size even if wild meat was shipped less often than domestic meat.

### Sale of wild and domestic meat (objective three)

Prices of wild and domestic meat are reported by meat-seller interviews in S2 Table and data from consumer interviews are in S4 Table. In accordance with hypothesis (3A), in 7 out of 9 cases where comparable purchases were made (same animal group, source type, and unit of purchase), the average price paid by consumers in urban areas was higher than that in rural areas (S4 Table); the exceptions were for purchases of wild pig (S4 Table).

In contrast to hypothesis (3B), according to respondents of the meat consumption interviews, prices varied by animal group but wild meat was less expensive overall (Nested mixed effects model; type of meat, F_1, 1613_ = 259.2, p < 0.0001; animal group [type of meat], F_5, 1611_ = 58.0, p < 0.0001; R^2^ = 0.61). Relative costs were only partially in line with expectations: bat meat was indeed less expensive than all domestic meat, but contrary to expectations, tenrec and wild pig meats generally were as well (S2 Fig).

In contrast to hypotheses (3C) and (3D), the cost of wild meat (as reported by respondents of the meat consumption interviews) did not differ depending on whether it was purchased from a hunter, in an open-air market, at a restaurant, or from a middleman (Mixed Effects Model, F_3, 35.6_ = 1.96, p = 0.14, model R^2^ = 0.33; S4 Table).

In accordance with hypothesis (3E), data taken from the meat-seller interviews indicates that, in open-air markets, the percentage of meat-sellers who had sold wild meat in the three days prior to the interview was lower (≤ 1%) than the percentage who had sold domestic meat (≥ 18%); S2 Table).

**Fig 8:**
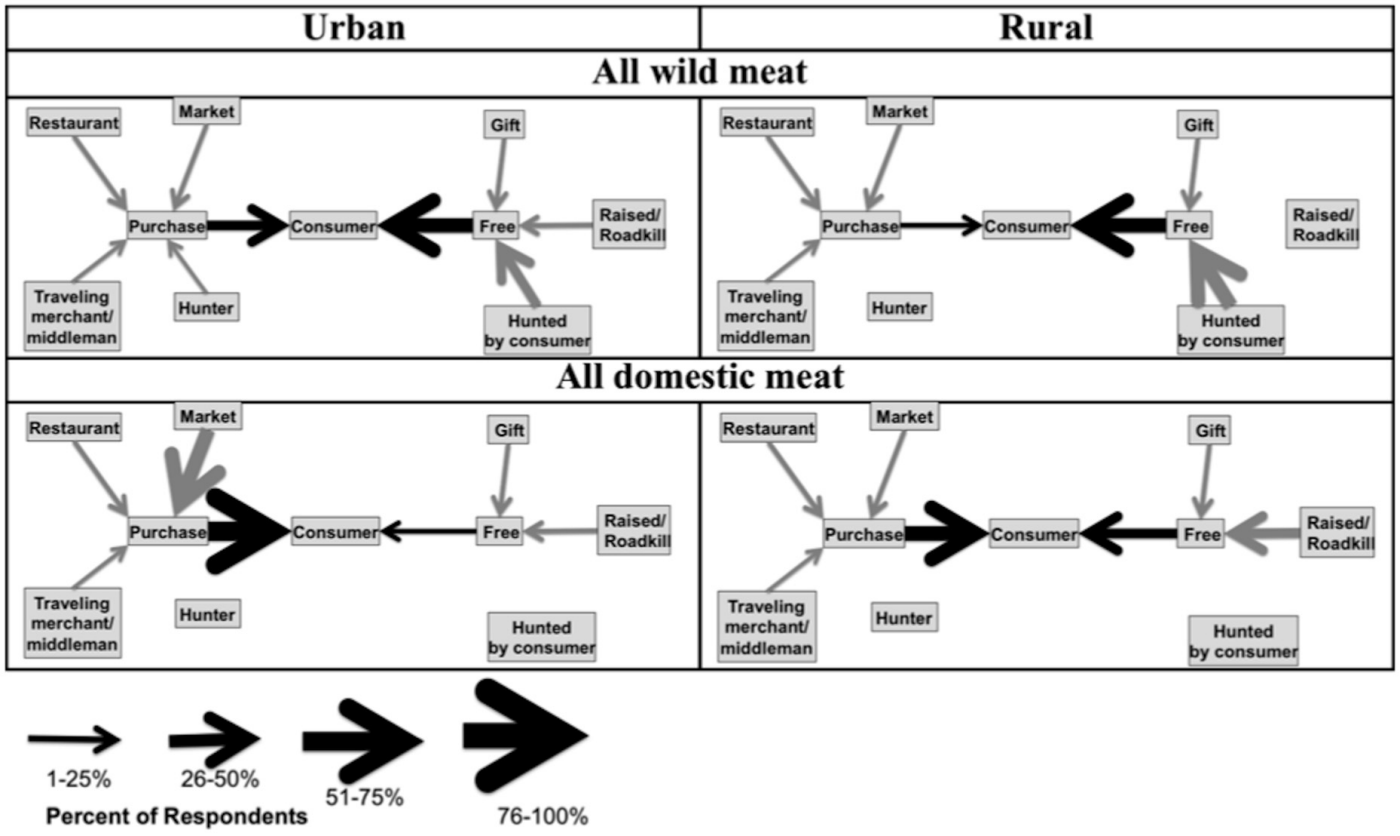
Patterns of movement for wild and domestic meats as reported by urban and rural respondents. The arrows point in the direction of the consumer and the thickness of the arrow is proportional to the percentage of consumers (towns are replicates) who procured domestic meat from that source. When no arrow is present, no respondents reported procuring meat from that source. There are two tiers of arrows; the arrows that connect peripheral boxes to the “purchase” and “free” boxes represent direct quantities measured (black arrows) while the arrows connecting the “purchase” and “free” boxes to the consumer represent sums of the peripheral boxes of each type (gray arrows).

In accordance with hypothesis (3F), and based on data taken from the meat consumption interviews, the patterns of movement of domestic and wild meat (S3, S4 and S5 Figs) from the source to the consumer may reflect their status as a legal, semi-legal (can be legally hunted some of the time), or illegal substance (legal domestic meats versus semi-legal and illegal wild meats, Fig 8). Domestic meat is often obtained through purchase or raised by the consumer rather than being provided as a gift (S5 Table), though urban respondents tend to purchase domestic meat from markets while (apart from zebu meat) rural respondents tend to own and raise domestic meat (S3 Fig). In contrast, the patterns of procurement for mammals that are commonly consumed as wild meat are less clear but include a variety of means including purchasing and hunting (S4 Fig). In further contrast, animals that are not commonly consumed or which are typically killed through human-wildlife conflict (e.g. carnivores that killed livestock or wild pigs that trampled rice fields) were almost exclusively consumed after being killed by the consumer (S5 Fig).

There was little support for hypothesis (3G). As reported in the meat consumption interviews, a higher percentage of respondents procured meat from free sources (e.g. by hunting or receiving the meat as a gift) than through purchase from a third party (S6 Table). The exception was that a higher percentage of urban respondents purchased bat and wild pig meat than procured it from free sources (S6 Table).

In contrast to hypothesis (3H), the population of a town had no effect on the percentage of consumers who bought wild meat (Regression, ln(population = 0.03, F_1,16_ = 1.88, p = 0.17, R^2^ = 0.01, towns as replicates with percentage of consumers arcsine-transformed).

### Volume of meat consumption (objective four)

The 1343 respondents to the meat consumption interviews provided 1046 estimates of yearly consumption rates for nine types of wild meat and 1282 estimates for three types of domestic meat. Most individuals had consumed wild meat at least once in their lifetimes (78 ± 7% across all respondents, 76 ± 9% of rural respondents, 80 ± 11% of urban respondents, towns as replicates); very few respondents were vegetarian (<1%).

Urban respondents estimated that they had consumed wild meat 86 ± 56 (mean ± 95% CI, towns as replicates) times in their lifetime and 10 ± 6 times per year in the recent past (from 2009–2013). Rural respondents estimated that they had consumed wild meat 117 ± 122 times in their lifetime and 5 ± 5 times per year in the recent past (S7 Table).

In accordance with hypotheses (4A) and (4B), the frequency of wild meat consumption per year differed substantially by animal group and domestic meat was consumed much more frequently per year than wild meat in this same period (Nested Mixed Effects Model; type of meat, F_1, 2269_ = 673.7, p < 0.0001; animal group [type of meat], F_9, 2261_ = 39.8, p < 0.0001; model R^2^ = 0.46). Specifically, the most-consumed meats were zebu, pig, and chicken (all domestic meats) in that order, followed by all the wild meats. Among wild meats it was clear that tenrec, wild pig, and bat were consumed more than civet or wild cat; lemur was also consumed more than wild cat. Consumption of fosa and mongoose relative to other wild meats could not be discerned (S6 Fig). Urban and rural regions consumed domestic meat an average of 51 ± 11 and 41 ± 17 times per year, respectively.

## Discussion

This is the first study to examine all stages of the commodity chain for wild meat in Madagascar, from hunter through transport, sale, and to the consumer. It is also the first study to compare aspects of the Malagasy wild meat trade across rural and urban areas, and to include both formal and informal enterprises. We also examined how the trade of wild meat differed by mammalian group and from domestic meats. Our findings suggest that, apart from the hunting stage, which was entirely informal, the commodity chain for wild meat in Madagascar is likely more formalized than previously thought. The characteristics of the stages in the commodity chain varied between wild meat and domestic meat, by wild meat type, and between urban and rural respondents. These results and their implications are discussed in more detail, below.

### Hunting and capture of wild meat

Similar to previous reports [19,20,35], a wide range of hunting methods were used to capture wild animals. The use of dogs while hunting was reported by a third of respondents, though hunting methods differed by animal group and between urban and rural respondents (Fig 3). For example, only urban respondents reported using blow tubes, firearms, cutting down trees to capture hollow-dwelling nocturnal lemurs, and digging up burrows to catch estivating tenrecs. These results are supported by anecdotal reports that the use of firearms in Madagascar to hunt lemurs is somewhat limited to those who can afford to own them. In two previously documented cases, firearms were loaned to hunters by third-party “entrepreneurs” and traders [19,36], while a third report noted that wealthier, urban individuals would go on weekend hunting trips using their rifles to hunt [37]. In eastern Madagascar a survey estimated that most lemurs were hunted with firearms, though the methods used may not have detected alternative hunting strategies, such as traps [26]. In addition to providing further evidence that lemurs are hunted using firearms, our data also reveal that this is just one of a number of methods used (Fig 3).

Our data confirm that hunting methods differ depending on intent and between urban and rural locations. For example, animals captured for sale were caught using more efficient methods than those captured for personal consumption (Fig 5) and the majority (93%) of animal captures for sale were by urban hunters. Therefore, it is reasonable to hypothesize that urban respondents might generally hunt for commercial reasons (although hunting for pleasure or when visiting family in rural areas may also be secondary motivators). In contrast, rural respondents might be more likely to hunt for subsistence [16] or to protect their agricultural lands and livestock from nuisance animals (wild pigs and carnivores) [15]. However, there are exceptions to these generalizations and rural respondents also do occasionally use more efficient hunting methods [19,20] and also sell their wild meat catches when they have caught surplus animals [16].

### Movement of wild animals away from the capture location

Many animal groups were hunted locally (<10 km) in rural areas, confirming observations from other studies [15]. However, we also found that the distance that respondents traveled to hunt wild meat increased with the population of a respondent’s town of residence. Urban individuals typically traveled farther than rural respondents to hunt wild meat (Fig 8A); the average distance traveled by urban individuals to hunt was always greater than 70 km from their town of residence (S1 Table).

The distances that commercial wild meat (wild meat that was purchased by the end consumer) traveled from the capture location to the consumer depended on the vendor type (Fig 7B, S1 Table). Also, the distance traveled by consumers to purchase wild meat increased with the population of a town. This makes sense given that consumers attempting to purchase wild meat might have to travel farther to areas where wild meat is sold (perhaps to regions closer to remnant habitat). Alternatively, this large travel distance may also be because some types of wild meat were consumed using expendable income (e.g. consumed for a special occasion) during an overnight visit with family members or on a business trip. While these distances are large—and while transporters reported moving wild meat up to 389 kilometers, on average—the commodity chain of Madagascar seems to cover the same distances as those examined in other areas of sub-Saharan Africa [4,10].

Comparing the transport patterns of wild meat to domestic meat can help highlight how the legal status of different wild meats may affect their commodity chains. Domestic meat was purchased more locally than wild meat in both urban and rural areas and was transported more regularly using inter-city transit systems. Despite transporting various domestic types of meats regularly, few drivers (2.7 ± 5.2%) had transported wild meat in the three days prior to the interview, and all who had, were from the northern-most town of Antsiranana. Furthermore, not all wild animal groups were transported using this system. Carnivores had never been transported by any of the drivers interviewed perhaps because they are generally caught due to human-wildlife conflict (interactions between people and wild animals resulting in negative impacts on people and their resources or on wild animals and their habitats [21,25]; these animals are legal to catch if they are a threat to agriculture or livestock, S1 Appendix). Lemurs were very rarely transported, perhaps because they are illegal to capture and hunt, and the illegal nature of these activities are relatively well known due to extensive outreach campaigns (focusing specifically on lemurs; e.g., the Lemur Action Plan). It is conceivable that inter-city transporters (who require permits to move certain types of meat) would not risk transporting a lemur (due to outreach campaigns surrounding this animal group) but would move other types of wild meat. By contrast, bats, tenrecs, and wild pigs—which are legal to capture and to sell under certain conditions and which were the most frequently consumed animals in our respondent pool (S7 Table)—were most frequently transported. These data suggest that: (1) animals like carnivores, that are often caught as a byproduct of human-wildlife conflict, are not typically moved far from the capture location; (2) some wild meats—like that from lemurs, which are illegal to hunt—are probably being moved from the capture location but potentially in private vehicles due to the illicit nature of the trade; and (3) some wild meats—like from bats, tenrecs, and wild pigs—are commonly moved via the country’s intercity transit system.

### Exchange of wild meat via sale, barter, or gifting

With the exception of urban respondents who consumed bat and wild pig meat, more respondents procured wild meat from free sources (e.g., by hunting or receiving the meat as a gift) than through purchase from a third party. However, many respondents did still purchase meat, and the percentage of consumers who purchased wild meat did not change with town population size.

Wild meat is typically consumed when it is a cheaper protein option than domestic meats [1,38]. We found that wild meat (with the exception of wild pig, which was priced similarly to zebu) was less expensive than domestic meat when portion sizes and venue of purchase were accounted for. This contrasts with prior studies in Madagascar, which reported that some types of wild meat were more expensive than domestic meat [21,23]. In addition, we found that wild meat was more expensive in urban areas than in rural areas but in contrast to other wild meat markets in Africa [4], wild meat did not differ in cost by type of venue (e.g., restaurant, market), when controlling for town, animal group, and unit of purchase.

In comparison to domestic meat, which was widely available, wild meat was sold at only a few open-air markets (S2 Table) and restaurants in each town. Usually, where these market stalls and restaurants were established enterprises, they were well known in the communities for their status as serving wild meat. Market stalls—regardless of whether they sold domestic or wild meat—usually specialized in the sale of one or two types (taxonomic groups) of meat. In contrast, restaurants—regardless of whether they sold wild meat or not—usually offered at least three different types of meat per week.

#### Consumption of wild meat

In accordance with prior studies [21,24,26] we found that wild meat was not consumed as frequently as domestic meat (S7 Table). However, we found that there were marked differences between urban and rural respondents in the consumption of wild meat; urban respondents consumed 24% more domestic meat per year and twice as much wild meat per year as their rural counterparts (S7 Table).

It is possible that the motivations for consuming wild meat differ between urban and rural populations. Urban consumers travel further to hunt than rural respondents, still hunt at similar levels to rural respondents, and purchase some meats (e.g. wild pig) with prices that are not always cheaper than domestic meats (and prices that are more expensive than those paid by rural respondents). We therefore hypothesize that consumption of wild meat in urban areas of Madagascar is largely driven by a preference for those meats. In contrast, we hypothesize that consumption in rural areas is driven by a mixture of preference for wild meats (rural respondents also purchase wild meat, though it is often cheaper than domestic meat), due to human-wildlife conflict (when animals, like carnivores, are captured in traps aimed at protecting livestock), or for subsistence. Our conclusions are consistent with other studies that suggest that the hunting and consumption of wild meat in rural Madagascar provides substantial economic value and food security to households [16,24] while in urban Madagascar, personal preference impacts consumption [21]. It is conceivable that the urban poor would also consume wild meat to fulfill their food security needs, a trend that has been seen elsewhere in sub-Saharan Africa [38]. However, in Madagascar, it is unlikely that these individuals would have the resources to travel to rural areas (where wild animals might still exist in remnant habitats) or sufficient financial capital to purchase wild meat in urban markets.

### Conservation implications

Our data indicate that the transport and exchange of wild meat in Madagascar are more commercial and more formalized than previously thought. They also highlight how interconnected and interdependent the informal and formal aspects of the commodity chain are; hunting appears to be mostly informal while other actors involved in the commodity chain include both formal and informal enterprises. Prior studies—which were based predominantly in rural areas of Madagascar and/or were limited to one geographic region—suggested that 98% of an individual’s wild meat was hunted by the consumer [16] and that hunting for wild meat is conducted for sustenance only [24]. However, our data indicate that while hunting is still an important source of wild meat, a large portion of wild meat passes through third-party actors. For example, urban respondents purchased 56% and 62% of the bats and wild pigs prior to consumption, while rural respondents purchased 28% and 31% of the bats and lemurs that they consumed (S6 Table). In addition, a significant portion of wild animals in Madagascar are moved across long distances using the intercity transit system and sold through venues known for their status as ‘wild meat selling establishments’. These venues include market stalls that specialize in selling wild meat (bats, tenrecs, and wild pigs) most days of the week. Given: (1) the distance traveled by consumers to purchase wild meat (S1 Table); (2) the well-known status of individual restaurants as ‘wild meat-selling establishments’ in each town where data was collected; and (3) that < 1% of meat-sellers at open-air markets sold wild meat in the three days prior to our interviews (S2 Table), it is reasonable to assume that the majority of the volume of wild meat trading in Madagascar is either hunted by a consumer [16,24] or sold through a small number of specialized market sellers and/or restaurants who are known to offer wild meat to consumers. The small number of dedicated wild meat-sellers may explain why prior researchers hypothesized that the Malagasy wild meat trade is not as formalized as in other areas of Africa [15]. However, these few dedicated meat-sellers may still be selling large amounts of wild meat and, similar to other wild meat commodity chains in sub-Saharan Africa [4], we found that the system of moving wild meat from rural areas to select urban venues using inter-city transport across large distances seems to be relatively organized. Together, the formal and informal commodity chain in Madagascar appears capable of moving at least thousands of wild meat carcasses per year at minimum from the point of capture to the final consumer (S3 Table).

Our data show that some animal groups face different threats from the wild meat trade than others. For example, while the consumption of bats decreased in both urban and rural areas following the 2009 coup d’état, meat-sellers indicated that they likely sold hundreds of bats per year; the threat to bats from the wild meat trade comes from both informal and illegal trading, as well as the formal and legal trade. It has been noted that awareness of wildlife laws protecting game species—including bats—is poor, and therefore they may not be effective [39]. In contrast, lemurs—which continue to be consumed in both rural and urban settings—are hardly ever traded through fixed venues (such as markets and restaurants) and it is therefore difficult to make any estimates regarding the volume of the trade of this animal group. We found little evidence for the luxury trade of lemurs which was widely reported following the coup d’état in 2009 [40]; we could not locate a single restaurant that currently sold lemur meat and most of the consumer reports of lemur consumption at restaurants were at least five years old. Therefore, we conclude that the threats facing lemurs from the wild meat trade likely do not typically involve restaurants and markets; instead, the threats come from the informal trade.

Understanding the commodity chain can help identify points along the chain for conservation interventions [4,5]. Conservation initiatives in Madagascar would need to have a broad focus in order to tackle sustainability issues during the first stage of the commodity chain, capture and hunting, since hunting is mostly informal (e.g. most hunters do not have hunting permits [26]), carried out by a variety of actors, and can be legal or illegal, depending on the animal group (S1 Appendix). In some cases, partial limits on hunting (such as restriction of methods or time periods in which hunting may take place) may be more difficult to enforce than outright bans, since the illegal nature of capture may not be evident at later stages, such as at the point of sale or consumption; this may thwart any enforcement efforts or confuse customers eager to follow the rules. In such cases, highly threatened species may benefit from hunting bans. It is unlikely that hunting bans alone will substantially reduce unsustainable hunting though [5], given that most hunting in Madagascar is done illegally already [16]. Any tightened restrictions would likely have to be paired with increased enforcement to be successful, but increasing enforcement would be difficult to mobilize given limited budgets and personnel and other pressing socio-economic priorities in Madagascar. If obstacles to increasing enforcement are too great, voluntary monitoring or citizen science programs could be instituted. For example, locally based monitoring programs in sub-Saharan Africa that worked with wild meat hunters to collect monitoring data, were found to be a cost-effective and accurate method of data collection [41]. At a minimum, these would help inform managers of current capture rates and help provide information for how programs could help influence the demand for wild meat at other points in the commodity chain. In addition, it can help inform community-based conservation programs [42] or Integrated Conservation and Development Programs [43]. For example, information collected from hunters and communities in northeast Madagascar has led to the development of alternative livelihoods programs which aim to decrease natural resource use within the boundaries of a protected area while providing hunters with more sustainable economic opportunities [2,15].

The second stage of the commodity chain—transportation—provides some opportunities for conservation programming. Some animals groups—including bats, tenrecs, and wild pigs—were often transported by inter-city transit (S3 Table) and were sometimes even delivered directly to restaurants for sale to the final consumer. These meats could be monitored using existing legal frameworks that require transporters and restaurants to have permits to move and sell wild meat (S1 Appendix), although enforcement and monitoring at transit hubs would need to be increased. Given that inter-city transit operations did not exclusively move wild meat (and wild meat transport did not constitute the major source of income for any driver interviewed), and already did not transport some illegal meats (like lemurs), drivers could be expected to cooperate with such a program. However, conservation initiatives would need to be prepared for the possibility that increased enforcement but unchanging consumer demand could increase the informal transportation of wild meat.

The third step of the commodity chain—the sale, barter, or gifting of wild meat—also presents some opportunities for conservation planning. We found that a high proportion of wild meat was sold through a few, well-known, fixed establishments, many located in urban areas. Targeting these relatively few establishments could present prime opportunities for the implementation of various management initiatives [4], including efforts to ensure compliance with existing laws and regulations governing harvest and sale of wild species. Animal groups—like bats, tenrecs, and wild pigs—that are traded through these formal enterprises could also be effectively monitored by working with meat-sellers using routine inspections. Food inspections are undertaken by the government in Madagascar, and may increase in the near future [44]; wild meat monitoring could be included in these inspections as a matter of course. Working with informal enterprises is more difficult but could involve restaurants and meat-sellers registering themselves as a wild meat-selling venue with different levels of voluntary and mandatory reporting requirements, depending on the types and quantity of wild meat being sold. Voluntary programs, where restaurants pledge not to sell certain types of foods (e.g. sustainable seafood; www.fish2fork.com) have been used by non-profit and multilateral groups in the past; these public pledges could be used in Madagascar to leverage the desire for tourists and other entities to support environmentally-friendly businesses. In addition, monitoring systems could provide early warning signals for the potential declines of wild populations; a permit system could be used to keep legal harvests sustainable and increased enforcement could be used to protect species that are not legal to sell.

Finally, the last step of the commodity chain—consumption by the consumer—presents opportunities for conservation programs aimed at curbing demand for species that are traded illegally or through informal venues, or where most of the hunting is conducted by the consumer. For these animal groups—like lemurs—public education and outreach might be the most effective way to decrease wild meat hunting consumption [45]. It has been noted that the Malagasy public has low knowledge of wildlife laws and only 43% of individuals could correctly classify different species into their legal categories (e.g. protected, game, and nuisance animals) [39]. Therefore, outreach to the public could include information about: (1) specific species and their legal status; (2) types of hunting methods that are prohibited; (3) seasons in which hunting is legal; and (4) permitting procedures. These outreach campaigns must consider that in urban areas, wild meat consumption in Madagascar may be driven by preference rather than by food security needs and that human-wildlife conflict may also be a factor contributing to hunting in rural areas. Therefore, successful outreach campaigns could appeal to the consumer in a way that acknowledges and leverages cultural and social norms—a tactic that has been successful in increasing community compliance in a marine reserve in southwest Madagascar [46]—while providing alternatives to individuals who are hunting mammals due to human-wildlife conflict [45] or for food security reasons [15]. Since compensation for damages caused by wildlife does not always succeed at protecting biodiversity, proposals to use compensation should be carefully considered prior to implementation [47], especially where funding sources needed to support such initiatives cannot be guaranteed over time. Finally, outreach in urban areas must be especially cognizant that, relative to rural consumers, urban consumers are willing to pay higher prices for wild meat, travel farther to procure wild meat, and utilize different hunting methods (such as firearms).

In some cases, there is no single best point along the commodity chain for conservation interventions [4]; it is important to consider all aspects of the commodity chain as targeted programming can have unintended consequences on other stages of the commodity chain [5]. For instance, the provision of alternative protein sources to communities can decrease hunter income and cause hunters to increase their hunting or to target higher value and possibly rarer species (to make up for lost income [5]). The political ecology of the commodity chain—the relationships and political agendas of different stakeholders and their impact on conservation programming—should also be considered as this can greatly impact the success of management and policy interventions [42]. To be effective, conservation programs will need to engage, empower, and support local communities [42]; when the local context is carefully considered and when appropriate frameworks and governance approaches are selected, conservation programming can succeed without sacrificing social development programming [48].

## Supporting Information

S1 Appendix The legality of hunting wild animals in Madagascar. (DOC)

S2 Appendix Interview materials. (DOC)

S1 Fig Percent of respondents who reported hunting wild mammal groups on a seasonal basis. Towns are replicates; data taken from meat consumption interviews.
(TIFF)

S2 Fig Cost per unit of wild versus domestic meats (A) and by animal group (B). In figure (B) types of meat on the right-hand side of the graph are domestic meats while those on the left are wild meats. Figure (B) shows least squares means of significant relationships between different animal groups calculated using a Tukey HSD post-hoc test; letters indicate significant differences.
(TIFF)

S3 Fig Sources of domestic meat in urban (left column) and rural regions (right column). The arrows point in the direction of the consumer and the thickness of the arrow is proportional to the percentage of consumers (towns are replicates) who procured domestic meat from that source. When no arrow is present, no respondents reported procuring meat from that source. There are two tiers of arrows; the arrows that connect peripheral boxes to the “purchase” and “free” boxes represent direct quantities measured (black arrows) while the arrows connecting the “purchase” and “free” boxes to the consumer represent sums of the peripheral boxes of each type (gray arrows).
(TIFF)

S4 Fig Sources of commonly consumed wild mammals in urban (left column) and rural regions (right column). The arrows point in the direction of the consumer and the thickness of the arrow is proportional to the percentage of consumers (towns are replicates) who procured wild meat from that source. When no arrow is present, no respondents reported procuring wild meat from that source. There are two tiers of arrows; the arrows that connect peripheral boxes to the “purchase” and “free” boxes represent direct quantities measured (black arrows) while the arrows connecting the “purchase” and “free” boxes to the consumer represent sums of the peripheral boxes of each type (gray arrows).
(TIFF)

S5 Fig Sources of meat for wild mammals not commonly consumed or killed through human-wildlife conflict. Sources of meat for wild mammals that are not commonly consumed or are typically killed through human-wildlife conflict in urban (left column) and rural regions (right column). The arrows point in the direction of the consumer and the thickness of the arrow is proportional to the percentage of consumers (towns are replicates) who procured domestic meat from that source. When no arrow is present, no respondents reported procuring meat from that source. There are two tiers of arrows; the arrows that connect peripheral boxes to the “purchase” and “free” boxes represent direct quantities measured (black arrows) while the arrows connecting the “purchase” and “free” boxes to the consumer represent sums of the peripheral boxes of each type (gray arrows).
(TIFF)

S6 Fig Impact of meat type on the frequency of yearly consumption of wild and domestic meats. The impacts of meat type on the frequency of yearly consumption by respondents between wild and domestic meats (A) and among different types of animal groups (B). In figure (B) types of meat on the right-hand side of the graph are domestic meats while those on the left are wild meats. Figure (B) shows least squares means of significant relationships between different animal groups calculated using a Tukey HSD post-hoc test. Letters indicate significant differences.
(TIFF)

S1 Table Average distances (in kilometers ± 95% CI, towns are replicates) traveled by the consumer to procure wild meat (no data from Rats/Mice). (DOCX)

S2 Table Sale of meat at open-air markets, restaurants, and supermarkets. Data are shown as the mean ± 95% CI (towns as replicates). Information taken from meat-seller interviews.
(DOCX)

S3 Table Meat transport via the bus and boat transport system (61 respondents across 5 urban towns). Data are shown as the mean ± 95% CI. Towns were used as replicates when sample sizes were higher (towns as replicates = TR) and individuals were used as replicates when sample sizes were low (individuals as replicates = IR).
(DOCX)

S4 Table Price paid by consumer for wild meat from different sellers (average, towns are replicates). All price estimates are from 2012 or 2013 unless otherwise noted. Body size estimate for wild cats was retrieved from Brockman et al. (2008). All other body size estimates were calculated from Garbutt (2007) as the mean of all species in the taxon, using the maximum weight recorded for each species.
(DOCX)

S5 Table Percent of people who procured domestic meat from different sources in urban and rural regions. Averages ± 95% CI are shown with towns as replicates.
(DOCX)

S6 Table Percent of people who procured wild meat from different sources in urban and rural regions. Averages ± 95% CI are shown and towns are replicates.
(DOCX)

S7 Table The volume of wild meat consumption. Includes individuals who had consumed wild meat at least once in a lifetime and the volume per year. Significant differences between towns are noted. Columns note whether towns or individual respondents are treated as replicates and depict the mean ± 95% Confidence Interval. Where individuals were used as replicates, sample sizes are listed behind each mean in parentheses. ND: No Data.
(DOCX)

## References

[1] Fa JE, Currie D, Meeuwig J. Bushmeat and food security in the Congo Basin: linkages between wildlife and people’s future. Environ Conserv J. 2003;30: 71–78.

[2] Golden CD, Fernald LCH, Brashares JS, Rasolofoniaina BJR, Kremen C. Benefits of wildlife consumption to child nutrition in a biodiversity hotspot. PNAS. 2011; 108:19653–19656. 10.1073/pnas.1112586108 22106297 PMC3241784

[3] Lindsey PA, Balme G, Becker M, Begg C, Bento C, Bocchino C, et al The bushmeat trade in African savannas: impacts, drivers, and possible solutions. Biol Conserv. 2013;160: 80–96.

[4] Cowlishaw G, Mendelson S, Marcus Rowcliffe J. Structure and operation of a bushmeat commodity chain in Southwestern Ghana. Conserv Biol. 2005; 19:139–149.

[5] Bowen-Jones E, Brown D, Robinson EJZ. Economic commodity or environmental crisis? An interdisciplinary approach to analyzing the bushmeat trade in Central and West Africa. Area. 2003;35: 390–402.

[6] Nasi R, Taber A, Van Vliet N. Empty forests, empty stomachs? Bushmeat and livelihoods in the Congo and Amazon Basins. International Forestry Review. 2011;13: 355–368.

[7] Bennett EL. Is there a link between wild meat and food security? Conserv Biol. 2002;16: 590–592.

[8] Crookes DJ, Ankudey N, Milner-Gulland EJ. The value of a long-term bushmeat market dataset as an indicator of system dynamics. Environ Conserv J. 2005;32: 333–339.

[9] Kumpel NF, Milner-Gulland EJ, Cowlishaw G, Rowcliffe JM. Incentives for hunting: the role of bushmeat in the household economy in rural Equatorial Guinea. Human Ecology. 2010;38: 251–264.

[10] Kamins AO, Restif O, Ntiamoa-Baidu Y, Suu-Ire R, Hayman DTS, Cunninham AA, et al Uncovering the fruit bat bushmeat commodity chain and the true extent of fruit bat hunting in Ghana, West Africa. Biol Conserv. 2011;144: 3000–3008. 22514356 10.1016/j.biocon.2011.09.003 PMC3323830

[11] Wilkie DS, Carpenter J. Bushmeat hunting in the Congo Basin: an assessment of impacts and options for mitigation. Biodivers Conserv. 1999;8: 927–955.

[12] Fa JE, Peres C. Hunting in tropical forests In: Reynolds JD, Mace GM, Redford KH, Robinson JG, editors. Conservation of exploited species. Cambridge: Cambridge University Press; 2001 pp. 203–241.

[13] Karesh WB, Cook RA, Bennett EL, Newcomb J. Wildlife trade and global disease emergence. Emerging Infectious Diseases. 2005;11: 1000–1002. 16022772 10.3201/eid1107.050194 PMC3371803

[14] Benjamin N, Mbaye AA. Informality, growth, and development in Africa: WIDER Working Paper 2014/052. Helsinki: World Institute for Development Economics Research; 2014.

[15] Golden CD. Bushmeat hunting and use in the Makira Forest, northeastern Madagascar: a conservation and livelihoods issue. Oryx. 2009;43: 386–392.

[16] Golden CD, Bonds MH, Brashares JS, Rasolofoniaina BJR, Kremen C. Economic valuation of subsistence harvest of wildlife in Madagascar. Conserv Biol. 2014;28: 234–243. 10.1111/cobi.12174 24405165 PMC4151980

[17] Cardiff SG, Ratrimomanarivo FH, Rembert G, Goodman SM. Hunting, disturbance and roost persistence of bats in caves at Ankarana, northern Madagascar. Afr J Ecol. 2009;47: 640–649.

[18] Schwitzer C, Mittermeier RA, Johnson SE, Donati G, Irwin M, Peacock H, et al Averting lemur extinctions amid Madagascar’s political crisis. Science. 2014;343: 842–843. 24558147 10.1126/science.1245783

[19] Vasey N. Clinging to life: Varecia variegate rubra and the Masoala Coastal Forests. Lemur News. 1996;2: 7–9.

[20] Goodman SM, Raselimanana A. Hunting of wild animals by Sakalava of the Menabe region: a field report from Kirindy-Mite. Lemur News. 2003;8: 4–6.

[21] Randrianandrianina FH, Racey PA, Jenkins RKB. Hunting and consumption of mammals and birds by people in urban areas of western Madagascar. Oryx. 2010;44: 411–415.

[22] Martinez B. Occurrence of Bamboo lemurs, Hapalemur griseus occidentalis, in an agricultural landscape on the Masoala Peninsula. Lemur News. 2008;13: 11–14.

[23] Tucker B. Applying behavioral ecology and behavioral economics to conservation and development planning: An example from the Mikea Forest, Madagascar. Human Nature. 2007;18: 190–208. 10.1007/s12110-007-9017-x 26181059

[24] Gardner CJ, Davies ZG. Rural bushmeat consumption within multiple-use protected areas: qualitative evidence from southwest Madagascar. Hum Ecol. 2014;42: 21–34.

[25] Razafimanahaka JH, Jenkins RKB, Andriafidison D, Randriananrianina F, Rakotomboavonjy V, Keane A, et al Novel approach for quantifying illegal bushmeat consumption reveals high consumption of protected species in Madagascar. Oryx. 2012;46: 584–592.

[26] Jenkins RKB, Keane A, Rakotoarivelo AR, Rakotomboavonjy V, Randrianandrianina FH, Razafimanahaka HJ, et al Analysis of patterns of bushmeat consumption reveals extensive exploitation of protected species in Eastern Madagascar. PLOS ONE. 2011;6: e27570 10.1371/journal.pone.0027570 22194787 PMC3237412

[27] Reuter KE, Gilles H, Wills AR, Sewall BJ. Live capture and ownership of lemurs in Madagascar: extent and conservation implications. Oryx. 2015: 10.1017/S003060531400074X

[28] Hawkins AF, Chapman AP, Ganzhorn JU, Blocam QMC, Barlow SC, Tonge SJ. Vertebrate conservation in Ankarana Special Reserve, northern Madagascar. Biol Conserv. 1990;54: 83–110.

[29] Ilo Program. Recensement des Communes 2001. 2003. Available: http://www.ilo.cornell.edu/ilo/data.html

[30] Rakotoarivelo AR, Razafimanahaka JH, Rabesihanaka S, Jones JPG, Jenkins RKB. Lois et règlements sur la faune sauvage à Madagascar: Progrès accomplis et besoins du future. Madag Conserv Dev. 2011;6: 37–44.

[31] Rietbergen-McCracken J, Narayan D. Participation and social assessment: tools and techniques. Washington, DC: World Bank; 1998.

[32] Reuter KE, Randell H, Wills AR, Sewall BJ. The consumption of wild meat in Madagascar: drivers, popularity, and food security. Environmental Conservation. In press.

[33] Golden CD, Wrangham RW, Brashares JS. Assessing the accuracy of interviewed recall for rare, highly seasonal events: the case of wildlife consumption in Madagascar. Animal Conservation. 2013;16: 597–603.

[34] United Nations. Operational Rates of Exchange. June 1st 2013. Available: http://treasury.un.org/operationalrates/OperationalRates.aspx

[35] Irwin MT, Smith TM, Wright PC. Census of three eastern rainforest sites north of Ranomafana National Park: preliminary results and implications for lemur conservation. Lemur News. 2000;5: 20–22.

[36] Duckworth JW, Evans MI, Hawkins AFA, Safford RJ, Wilkinson RJ. The lemurs of Marojejy Strict Nature Reserve, Madagascar: A status overview with notes on ecology and threats. Int J Primatol. 1995;16: 545–559.

[37] Rigamonti MM. Red ruffed lemur (Varecia variegata rubra): A rare species from the Masoala Rain Forests. Lemur News. 1996;2: 9–11.

[38] van Vliet N, Nebesse C, Gambalemoke S, Akaibe D, Nasi R. The bushmeat market in Kisangani, Democratic Republic of Congo: implications for conservation and food security. Oryx. 2012;46(2): 196–203.

[39] Keane A, Ramarolahy AA, Jones JPG, Milner-Gulland EJ. Evidence for the effects of environmental engagement and education on knowledge of wildlife laws in Madagascar. Conserv Lett. 2011;4: 55–63.

[40] Barrett MA, Ratsimbazafy J. Luxury bushmeat trade threatens lemur conservation. Nature. 2009;461: 470. 10.1038/461470a 19779432

[41] Rist J, Milner-Gulland EJ, Cowlishaw G, Rowcliffe M. Hunter reporting of catch per unit effort as a monitoring tool in a bushmeat-harvesting system. Conserv Biol. 2010;24: 489–499. 10.1111/j.1523-1739.2010.01470.x 20491849

[42] Berkes F. Rethinking Community-based conservation. Conserv Biol. 2004;18(3): 621–630.

[43] Guerbois C, Dufour A-B, Mtare G, Fritz H. Insights for integrated conservation from attitudes of people towards protected areas near Hwange National Park, Zimbabwe. Conserv Biol. 2013;27: 844–855. 10.1111/cobi.12108 23866038

[44] Sarter S, Sarter G, Gilabert P. A swot analysis of HACCP implementation in Madagascar. Food Control. 2010;21: 253–259.

[45] Breuer T, Mavinga FB. Education for the conservation of great apes and other wildlife in Northern Congo—the importance of nature clubs. Am J Primatol. 2010;72: 454–461. 10.1002/ajp.20774 19937737

[46] Westerman K, Gardner CJ. Adoption of socio-cultural norms to increase community compliance in permanent marine reserves in southwest Madagascar. Conservation Evidence. 2013;10: 4–9.

[47] Bulte E, Rondeau D. Compensation for wildlife damages: habitat conversion, species preservation and local welfare. J Environ Econ Manage. 2007;54: 311–322.

[48] Campbell BM, Sayer JA, Walker B. Navigating trade-offs: working for conservation and development outcomes. Ecol Soc. 2010; 15:16.

