# Supplementary material for: Capture, Movement, Trade, and Consumption of Mammals in Madagascar
Source: PLoS One. 2016 Feb 29;11(2):e0150305. doi: 10.1371/journal.pone.0150305 (PMC4771166; doi:10.1371/journal.pone.0150305)
Supplement: S1 Appendix — (DOC) [file pone.0150305.s001.doc]

**Appendix S1**

*The legality of hunting wild animals in Madagascar:*

In Madagascar, the legality of hunting wild animals is dictated through a three-tier classification system. Animals are classified into three categories: 1) protected (can never be hunted), 2) harmful and nuisance animals (can be hunted year-round; animals which damage crops or kill domestic animals); and 3) game animals (hunted during a multi-month hunting season that is set yearly by the government; Rakotoarivelo et al. 2011). Some methods of hunting are prohibited in Madagascar. For example, it is never legal to hunt at night, to use poisoned bait or sedatives, explosives, with fire (Rakotoarivelo et al. 2011), or by using nets and pit traps (Ordonnance nº 60-126). Hunting with “weapons of local manufacture” including spears, bows, and blowguns are authorized, but only for non-commercial purposes (Ordonnance nº 60-126) and when permitted (Rakotoarivelo et al. 2011). Extra permits are needed to for the use of firearms and for commercial and scientific hunting (Rakotoarivelo et al. 2011).

As in many African countries (Lindsey et al. 2013), hunting in Madagascar is regulated using legal instruments, with the right to hunt belonging to the state and allowed through a permit-based hunting system (Rakotoarivelo et al. 2011). The same regulations which apply to hunters also apply to the “transport, peddling, sale, purchase, and release for consumption (of wild meat in) inns or restaurants” (Ordonnance nº 60-128). Government officials, district-level officials, army officers, judicial police, customs officers, service agents of livestock, and managers of food halls and markets, all have the right to, “search and recognize offenses” of illegally acquired wild animals (Ordonnance nº 60-128). These individuals have the authority to enter storage rooms, kitchens, offices, and other private or public areas relevant to an innkeeper’s or restaurant’s trade, as it relates to the trade of animals and they can: 1) seize gear and firearms used in the hunting offense; and 2) sequester motor vehicles (cars and boats) used to hunt or transport animals (Ordonnance nº 60-128). Punishment for being caught with illegal wild meat includes: 1) a fine of 10,000 to 200,000 Ariary (5 to 100 USD; for comparison, 81.3% of the population lives on < 1.25 USD per day, UNDP 2013); 2) imprisonment for one month to two years; and 3) loss of hunting license and/or authorization to hunt commercially (Ordonnance nº 60-128). Offenders will always have weapons, gear, and vehicles confiscated and will always be imprisoned if it is the second offense within five years (Ordonnance nº 60-128). Individuals who transport, peddle, sell, export, or store animals in violation of regulations are also liable to the same punishments as the individuals who hunted the animal; this includes restaurants that store the meat and serve it to customers (Ordonnance nº 60-128). Defendants cannot claim “ignorance of zoological matters” to justify violating the law (Ordonnance nº 60-128).

*Literature cited:*

Lindsey, P.A., G. Balme, M. Becker, C. Begg, C. Bento, C. Bocchino, A. Dickman, R. W. Diggle, H. Eves, P. Henschel, D. Lewis, K. Marnewick, J. Mattheus, J. Weldon McNutt, R. McRobb, N. Midlane, J. Milanzi, R. Morley, M. Murphree, V. Opyene, J. Phadima, G. Purchase, D. Rentsch, C. Roche, J. Shaw, H. van der Westhuizen, N. Van Vliet, and P. Zisadza-Gandiwa. 2013. The bushmeat trade in African savannas: impacts, drivers, and possible solutions. Biological Conservation **160**:80-96.

Ordonnance nº 60-126: fixant le régime de la chasse, de la pêche et de la protection de la faune. October 3 1960. Republic of Madagascar. Available online via the Food and Agricultural Organization of the United Nations Legal Office: <http://faolex.fao.org/docs/pdf/mad4214.pdf> Accessed March 24 2015

Ordonnance nº 60-128: fixant la procédure applicable à la répression des infractions à la législation forestière, de la chasse, de la pêche et de la protection de la nature, modifiée par l'ordonnance nº 62-085. September 29 1962. Available online via the Food and Agricultural Organization of the United Nations Legal Office: <http://faolex.fao.org/docs/pdf/mad2819.pdf> Accessed March 24 2015

Rakotoarivelo, A. R., J. H. Razafimanahaka, S. Rabesihanaka, J. P. G. Jones, and R. K. B. Jenkins. 2011. Lois et règlements sur la faune sauvage à Madagascar: Progrès accomplis et besoins du future. Madagascar Conservation and Development **6**:37-44.

United Nations Development Programme [UNDP]. 2013. Human development report 2013: The rise of the south: Human progress in a diverse world. United Nations Development Programme, New York, USA.
