# Supplementary material for: Capture, Movement, Trade, and Consumption of Mammals in Madagascar
Source: PLoS One. 2016 Feb 29;11(2):e0150305. doi: 10.1371/journal.pone.0150305 (PMC4771166; doi:10.1371/journal.pone.0150305)
Supplement: S2 Appendix — (DOC) [file pone.0150305.s002.doc]

**Appendix S2**

SURVEY:

Section One:

*In this section, respondents were asked about their wild meat and domestic meat consumption. Interviewers reminded respondents that we were interested in all kinds of meat consumed, including gifts, wild meat, purchased meat, and farmed/domestic meat.*

1. Please list all of the different meats, not including fish, which you have eaten in the past three days. *The following follow-up questions were asked for each meat named:*
   1. How did you get this meat? (*Answer choices: Purchase, Caught, Gift, Raised*)
   2. *If purchased:* How much did it cost? Where did you purchase it?
   3. *If caught*: Where and when did you catch it?
2. Please list your top five favorite types of meat, not including fish. *The following follow-up questions were asked for each meat listed:*
   1. How often do you get to eat it? (*We encouraged respondents to provide weekly or monthly estimates of consumption frequencies*)
   2. Do you usually buy it? For what price? Where do you buy it?
   3. Do you usually catch it? Where do you catch it?
   4. Is this something you eat for a special occasion? Why do you consume this meat?

Section Two:

1. Have you ever eaten a tenrec before? *If yes, the follow-up questions were asked.*
   1. How often do you get to eat it per year? How often have you eaten it in your lifetime? (*We encouraged respondents to provide weekly or monthly estimations of consumption frequencies if they could not provide a yearly estimate*)
   2. When did you last eat it?
   3. Do you usually buy it? For what price? Where do you buy it?
   4. Do you usually catch it? Where do you catch it?
   5. Is this something you eat for a special occasion? Why do you consume this meat?
2. Have you ever eaten bat before? (*See question 3 for follow-up questions*)
3. Have you ever eaten fossa before? (*See question 3 for follow-up questions*)
4. Have you ever eaten mongoose before? (*See question 3 for follow-up questions*)
5. Have you ever eaten rats and mice before? (*See question 3 for follow-up questions*)
6. Have you ever eaten civets before? (*See question 3 for follow-up questions*)
7. Have you ever eaten wild cats before? (*See question 3 for follow-up questions*)
8. Have you ever eaten wild pigs before? (*See question 3 for follow-up questions*)
9. Have you ever eaten lemur before? (*See question 3 for follow-up questions*)

Section Three:

1. Have you changed the type of meat that you eat? If yes, how? If yes, why?

*Example: did you formerly eat a lot of zebu, but now you eat fish? Or did you formerly eat a lot of wild meat, but now you eat chicken?*

1. Do you have any taboos (fady) about eating any animals in your area? Who follows these fadys (an individual family, ethnicity, or the whole village?)? What are the taboos?
