# Supplementary material for: Capture, Movement, Trade, and Consumption of Mammals in Madagascar
Source: PLoS One. 2016 Feb 29;11(2):e0150305. doi: 10.1371/journal.pone.0150305 (PMC4771166; doi:10.1371/journal.pone.0150305)
Supplement: S1 Table — (DOCX) [file pone.0150305.s009.docx]

**Table S1.** **Average distances (in kilometers ± 95% CI, towns are replicates) traveled by the consumer to procure wild meat (no data from Rats/Mice).**

|  | **Free** | | | **Purchased** | | | |
| --- | --- | --- | --- | --- | --- | --- | --- |
| **Animal Group** | **Hunted animal** | **Roadkill/Raised** | **Gift** | **From hunter** | **From middleman**** | **From restaurant** | **From market** |
| **Bat** |  |  |  |  |  |  |  |
| *Urban* | 135 ± 92 | --- | 126 ± 94 | 171 ± 334 | 0 (10 ± 10) | 147 ± 117 | 35 ± 26 |
| *Rural* | N.D. | --- | 122 (n=1 town) | --- | 0 (5, n =1 town) | 222 ± 100 | --- |
| **Civet** |  |  |  |  |  |  |  |
| *Urban* | 71 ± 9 | 0 ± 0 | 69 ± 102 | --- | 0 | --- | --- |
| *Rural* | 3 ± 3 | --- | 113 ± 221 | --- | 0 (0 ± 0) | --- | --- |
| **Fossa** |  |  |  |  |  |  |  |
| *Urban* | 177 ± 303 | --- | 16 ± 33 | --- | --- | 354 (n=1 town) | --- |
| *Rural* | 52 ± 75 | --- | 5 (n=1 town) | --- | 0 | --- | --- |
| **Lemurs** |  |  |  |  |  |  |  |
| *Urban* | 105 ± 78 | 0 ± 0 | 137 ± 105 | N.D. | 0 (101± 110) | 453 ± 545 | 0 (n=1 town) |
| *Rural* | 7 ± 5 | --- | 96 ± 188 | --- | 0 (7 ± 6) | 165 (n=1 town) | --- |
| **Mongoose** |  |  |  |  |  |  |  |
| *Urban* | 92 ± 105 | --- | 5 (n=1 town) | --- | --- | --- | --- |
| *Rural* | 160 ± 77 | --- | 250 ± 489 | --- | 0 (0, n=1 town) | --- | --- |
| **Tenrec** |  |  |  |  |  |  |  |
| *Urban* | 76 ± 31 | 0 ± 0 | 98 ± 57 | 0 ± 0 | 0 (22 ± 28) | 477 ± 359 | 129 ± 173 |
| *Rural* | 5 ± 4 | --- | 2 ± 3 | --- | 0 (0 ± 0) | 155 ± 108 | 100 km |
| **Wild Cat** |  |  |  |  |  |  |  |
| *Urban* | 89 ± 38 | 0 ± 0 | 117 ± 199 | N.D. | N.D. | N.D. | N.D. |
| *Rural* | 0 ± 0 | --- | N.D. | --- | --- | --- | --- |
| **Wild Pig** |  |  |  |  |  |  |  |
| *Urban* | 112 ± 159 | --- | 102 ± 90 | --- | 0 (166 ± 271) | 119 ± 197 | 198 ± 333 |
| *Rural* | <1 ± 1 | --- | 0 (n=1 town) | --- | 0 (4 ± 4) | --- | --- |

**Middlemen typically travel to a consumer’s town of residence, and the travel distances of the middleman to the consumer – as reported by the consumers – are listed in parentheses when available.
