# Supplementary material for: Capture, Movement, Trade, and Consumption of Mammals in Madagascar
Source: PLoS One. 2016 Feb 29;11(2):e0150305. doi: 10.1371/journal.pone.0150305 (PMC4771166; doi:10.1371/journal.pone.0150305)
Supplement: S2 Table — Data are shown as the mean ± 95% CI (towns as replicates). Information taken from meat-seller interviews. (DOCX) [file pone.0150305.s010.docx]

**Table S2. Sale of meat at open-air markets, restaurants, and supermarkets.**

| **Animal Group** | **Had ever sold (%)** | **Sold last 3 days (%)** | **Length of time since last sale (years ago)** | **Amount sold per day when selling type of meat** | **Percent of respondents who bought meat (distance traveled for purchase)** | **Percent of respondents who hunted meat (distance traveled to hunt)** | **Price of Purchase (Ariary)** | **Price of Sale (Ariary)** |
| --- | --- | --- | --- | --- | --- | --- | --- | --- |
| **Wild meat** |  |  |  |  |  |  |  |  |
| ***Bats***  *Markets*  *Restaurants*  *Supermarkets* | 1 ± 2  66 ± 36  0 ± 0 | 1 ± 2  54 ± 33  0 ± 0 | 2 ± 4  <1 ± <1  --- | 11 ± 14 animals  11 ± 5 plates  --- | 100 (0 ± 0)  100 (14 ± 27)  --- | ND  0  --- | 2812/Animal  4076/Animal  --- | 4666/Plate  5791/Plate  --- |
| ***Lemurs***  *Markets*  *Restaurants*  *Supermarkets* | 0 ± 0  2 ± 4  0 ± 0 | 0 ± 0  0 ± 0  0 ± 0 | ---  0 (n=1)  --- | ---  ND  --- | ---  ND  --- | ---  ND  --- | ---  5000/Animal  --- | ---  2000/Plate  -- |
| ***Tenrecs***  *Markets*  *Restaurants*  *Supermarkets* | <1 ± <1  29 ± 39  0 ± 0 | 0 ± 0  0 ± 0  0 ± 0 | 1 ± 0  <1 ± <1  --- | 19 ± 25 animals  7 ± 4 plates  --- | 100 (10 ± 20)  100 (32 ± 42)  --- | 0  0  --- | 5000/Animal  6800/Animal  --- | 6000/Animal  8400/Plate  --- |
| ***Wild Pigs***  *Markets*  *Restaurants*  *Supermarkets* | 3 ± 4  18 ± 20  100 | 1 ± 2  27 ± 27  66% | 2 ± 3  1 ± 2  <1 ± 1 | ND  7 ± 6 plates  ND | 75 (42 ± 40)  91 (0 ± 0)  67 (0 ± 0) | 25 (20 ± 0)  8 (92, n = 1)  33 (552, n =1) | 42500/Animal 4000/kg  3500/kg  ND | 4125/kg  13500/kg 15357/Plate  14716/kg |
| **Domestic Meat** |  |  |  |  |  | **% Raised animals** |  |  |
| ***Chicken***  *Markets*  *Restaurants* | ND | 18 ± 10  81 ± 26 | 0 ± 0  0 ± 0 | 7 ± 5 animals  5 ± 1 plates | 15±10 (54±42)  53±9 (0±0) | 4 ± 4  0 ± 0 | 6361/Animal  10,792/Animal 8575/kg | 12250/Animal  ND |
| ***Pig***  *Markets*  *Restaurants* | ND | 28 ± 12  18 ± 23 | 0 ± 0  0 ± 0 | 42 ± 37 kilograms <1 ± <1 animals  4 ± 1 plates | 24±11 (11±9)  18±23 (0±0) | 4 ± 4  0 ± 0 | 499,899/Animal 6575/kg  19,500/kg | 6466/kg  10,000/Plate |
| ***Zebu***  *Markets*  *Restaurants* | ND | 54 ± 13  46 ± 32 | 0 ± 0  0 ± 0 | 41 ± 14 kilograms <1 ± <1 animals  11 ± 10 plates | 51±13 (42±22)  43±32 (0±0) | 2 ± 4  0 ± 0 | 621,363/Animal 6009/kg  8479/kg | 140,000/Animal  8373/kg  9923/Plate |

Data are shown as the mean ± 95% CI (towns as replicates). Information taken from meat-seller interviews.
