# Supplementary material for: Capture, Movement, Trade, and Consumption of Mammals in Madagascar
Source: PLoS One. 2016 Feb 29;11(2):e0150305. doi: 10.1371/journal.pone.0150305 (PMC4771166; doi:10.1371/journal.pone.0150305)
Supplement: S3 Table — Data are shown as the mean ± 95% CI. Towns were used as replicates when sample sizes were higher (towns as replicates = TR) and individuals were used as replicates when sample sizes were low (individuals as replicates = IR). (DOCX) [file pone.0150305.s011.docx]

**Table S3. Transport of meat on the in-country bus and boat transport system (61 respondents across 5 urban towns).**

| Animal Group | Percent of drivers who had transported before (%) | How recently did you transport (years ago) | When transporting, how many are transported per trip? | How many do you transport per year? | Cost of transporting meat | Transport distance (km) |
| --- | --- | --- | --- | --- | --- | --- |
| Wild meat | *TR* | *IR* | *IR* | *IR* | *IR* | *IR* |
| *Bats* | 24 ± 27 | <1 ± <1 | 32 ± 4 | 1542 ± 954 | 4083 ± 2061 Ariary/Bag* | 389 ± 303 |
| *Lemurs* | 2 ± 4 | <1 ± <1 | ND | ND | 1000 ± 1959 Ariary/Animal | 36 ± 41 |
| *Tenrecs* | 12 ± 11 | 1 ± 2 | 10 ± 8 | 118 ± 73 | 100 ± 196 Ariary/Trip | 106 ± 79 |
| *Wild Cat* | <1 ± 1 | 1 (n = 1) | ND | ND | 0 (n = 1) Ariary/Animal | ND |
| *Wild Pigs* | 17 ± 18 | <1 ± <1 | 4 ± 4 Animals  30 ± 5 kg | 141 ± 269 Animals  2495 ± 1865 kg | 13928 ± 8544  Ariary/Animal | 128 ± 85 |
| Domestic Meat | *TR* | *TR* | *TR* | *TR* | *TR* | *TR* |
| *Chicken* | 62 ± 24 | ND | 27 ± 16 | ND | 444 ± 109 Ariary/Animal  7650 ± 5433 Ariary/Basket** | 223 ± 117 |

Data are shown as the mean ± 95% CI. Towns were used as replicates when sample sizes were higher (towns as replicates = TR) and individuals were used as replicates when sample sizes were low (individuals as replicates = IR).

* One bag reportedly holds anywhere from 10-60 bats.
** One basket can hold anywhere from 10-60 chickens, depending on the size.
