# Supplementary material for: Capture, Movement, Trade, and Consumption of Mammals in Madagascar
Source: PLoS One. 2016 Feb 29;11(2):e0150305. doi: 10.1371/journal.pone.0150305 (PMC4771166; doi:10.1371/journal.pone.0150305)
Supplement: S4 Table — All price estimates are from 2012 or 2013 unless otherwise noted. Body size estimate for wild cats was retrieved from Brockman et al. (2008). All other body size estimates were calculated from Garbutt (2007) as the mean of all species in the taxon, using the maximum weight recorded for each species. (DOCX) [file pone.0150305.s012.docx]

**Table S4.** **Price paid by consumer for wild meat from different sellers (average, towns are replicates).**

| **Animal Group** | **Purchase Price (Ariary)** | | | | |
| --- | --- | --- | --- | --- | --- |
|  | **All Sources** | **Hunter** | **Middleman** | **Market** | **Restaurant** |
| **Bats (0.12 ± 030 lbs)** |  |  |  |  |  |
| *Urban* | 3042/animal  2908/plate  ---  --- | 2500/animal  --- --- --- | 3044/animal  ---  ---  --- | 3550/animal  --- --- --- | 4000/animal  2761/plate ---  --- |
| *Rural* | 1798/animal  1750/plate  2500/serving  --- | --- | 1950/animal  ---  2500/serving  --- | 3136/animal --- --- --- | ---  2625/plate --- --- |
| **Civet (7.64 ± 3.04 lbs)** |  |  |  |  |  |
| *Urban* | 2150/animal 4000/serving | --- 4000/serving | --- | --- | --- |
| *Rural* | --- | --- | --- | --- | --- |
| **Fossa (price estimate from 2010) (22 lbs)** |  |  |  |  |  |
| *Urban* | 1000/plate | --- | --- | --- | 1000/plate |
| *Rural* | --- | --- | --- | --- | --- |
| **Lemurs (3.64 ± 4.18 lbs)** |  |  |  |  |  |
| *Urban* | 3000/animal  3000/plate  2125/serving  3000/kg | --- | 4625/animal  ---  2125/serving  3000/kg | --- | ---  3000/plate  ---  --- |
| *Rural* | 4667/animal  ---  1500/serving  --- | --- | 1000/animal  ---  1500/serving  --- | --- | --- |
| **Mongoose (undated price estimate) (2.04 ± 0.71 lbs)** |  |  |  |  |  |
| *Urban* | --- | --- | --- | --- | --- |
| *Rural* | 3000/animal | --- | --- | --- | --- |
| **Tenrecs (0.26 ± 0.80 lbs)** |  |  |  |  |  |
| *Urban* | 3298/animal  2000/plate  ---  6000/kg | 2000/animal  --- --- --- | 3358/animal  2000/plate  ---  --- | 4300/animal  --- --- --- | 3000/animal  --- ---  --- |
| *Rural* | 3000/animal  ---  ---  --- | --- | ---  ---  ---  --- | --- | --- |
| **Wild Cat (12 lbs)** |  |  |  |  |  |
| *Urban* | 2000/animal | --- | --- | --- | --- |
| *Rural* | --- | --- | --- | --- | --- |
| **Wild Pig (155 lbs)** |  |  |  |  |  |
| *Urban* | ---  7444/plate  3081/serving  4251/kg | --- | ---  ---  2279/serving  3466/kg | --- --- 2417/serving 4313/kg | ---  7444/plate  ---  --- |
| *Rural* | 30,000/animal  ---  2375/serving  4000/kg | --- | 30,000/animal  ---  2816/serving  4000/kg | --- | --- |

All price estimates are from 2012 or 2013 unless otherwise noted. Body size estimate for wild cats was retrieved from Brockman et al. (2008). All other body size estimates were calculated from Garbutt (2007) as the mean of all species in the taxon, using the maximum weight recorded for each species.
