# Supplementary material for: Capture, Movement, Trade, and Consumption of Mammals in Madagascar
Source: PLoS One. 2016 Feb 29;11(2):e0150305. doi: 10.1371/journal.pone.0150305 (PMC4771166; doi:10.1371/journal.pone.0150305)
Supplement: S5 Table — Averages ± 95% CI are shown with towns as replicates. (DOCX) [file pone.0150305.s013.docx]

**Table S5.** **Percent of people who procured domestic meat from different sources in urban and rural regions.**

|  | **Free (%)** | | | **Purchased (%)** | | | | |
| --- | --- | --- | --- | --- | --- | --- | --- | --- |
| **Animal Group** | **Consumer hunted animal** | **Consumer raised animal/**  **roadkill** | **Consumer received animal as gift** | **From all sources** | **From farmer** | **From middleman** | **From restaurant** | **From market/**  **supermarket** |
| **Chicken** |  |  |  |  |  |  |  |  |
| *Urban* | NA | 32 ± 9 | 3 ± 4 | 69 ± 10 | 0 ± 0 | 5 ± 5 | 2 ± 2 | 36 ± 16 |
| *Rural* | NA | 60 ± 15 | 3 ± 3 | 45 ± 13 | 0 ± 0 | 16 ± 12 | 2 ± 3 | 1 ± 2 |
| **Pig** |  |  |  |  |  |  |  |  |
| *Urban* | NA | <1 ± <1 | 0 ± 0 | 99 ± 1 | 9 ± 17 | 3 ± 6 | 1 ± 1 | 58 ± 25 |
| *Rural* | NA | 39 ± 30 | 22 ± 20 | 44 ± 31 | 0 ± 0 | 13 ± 17 | 0 ± 0 | 0 ± 0 |
| **Zebu** |  |  |  |  |  |  |  |  |
| *Urban* | NA | 0 ± 0 | 4 ± 3 | 96 ± 3 | 0 ± 0 | <1 ± 1 | <1 ± <1 | 62 ± 24 |
| *Rural* | NA | 2 ± 3 | 6 ± 3 | 94 ± 3 | 0 ± 0 | 14 ± 8 | 2 ± 3 | 5 ± 5 |

Averages ± 95% CI are shown with towns as replicates.
