# Supplementary material for: Capture, Movement, Trade, and Consumption of Mammals in Madagascar
Source: PLoS One. 2016 Feb 29;11(2):e0150305. doi: 10.1371/journal.pone.0150305 (PMC4771166; doi:10.1371/journal.pone.0150305)
Supplement: S6 Table — Averages ± 95% CI are shown and towns are replicates. (DOCX) [file pone.0150305.s014.docx]

**Table S6.** **Percent of people who procured wild meat from different sources in urban and rural regions.**

|  | **Free (%)** | | | **Purchased (%)** | | | | |
| --- | --- | --- | --- | --- | --- | --- | --- | --- |
| **Animal Group** | **Consumer hunted animal** | **Consumer raised animal/**  **roadkill** | **Consumer received animal as gift** | **From all sources** | **From hunter** | **From middleman** | **From restaurant** | **From market** |
| **Bat** |  |  |  |  |  |  |  |  |
| *Urban* | 28 ± 8 | 0 ± 0 | 21 ± 9 | 56 ± 13 | 2 ± 2 | 17 ± 13 | 10 ± 6 | 10 ± 9 |
| *Rural* | 46 ± 25 | 0 ± 0 | 11 ± 11 | 28 ± 16 | <1 ± <1 | 8 ± 7 | 4 ± 6 | 0 ± 0 |
| **Civet** |  |  |  |  |  |  |  |  |
| *Urban* | 67 ± 9 | 13 ± 6 | 18 ± 7 | 3 ± 2 | 0 ± 0 | <1 ± <1 | 0 ± 0 | 0 ± 0 |
| *Rural* | 59 ± 25 | 0 ± 0 | 18 ± 15 | 6 ± 5 | 0 ± 0 | 6 ± 8 | 0 ± 0 | 0 ± 0 |
| **Fossa** |  |  |  |  |  |  |  |  |
| *Urban* | 83 ± 18 | 0 ± 0 | 10 ± 14 | 5 ± 10 | 0 ± 0 | 0 ± 0 | 5 ± 10 | 0 ± 0 |
| *Rural* | 50 ± 31 | 0 ± 0 | 26 ± 23 | 7 ± 13 | 0 ± 0 | 8 ± 14 | 0 ± 0 | 0 ± 0 |
| **Lemurs** |  |  |  |  |  |  |  |  |
| *Urban* | 43 ± 13 | <1 ± <1 | 37 ± 10 | 15 ± 9 | 0 ± 0 | 9 ± 9 | 2 ± 2 | <1 ± <1 |
| *Rural* | 44 ± 22 | 0 ± 0 | 14 ± 12 | 31 ± 17 | 0 ± 0 | 19 ± 13 | 1 ± 2 | 0 ± 0 |
| **Mongoose** |  |  |  |  |  |  |  |  |
| *Urban* | 100 ± 0 | 0 ± 0 | 31 ± 23 | 0 ± 0 | 0 ± 0 | 0 ± 0 | 0 ± 0 | 0 ± 0 |
| *Rural* | 86 ± 28 | 0 ± 0 | 26 ± 30 | 14 ± 28 | 0 ± 0 | 17 ± 28 | 0 ± 0 | 0 ± 0 |
| **Rats/Mice** |  |  |  |  |  |  |  |  |
| *Urban* | 33% (n=1) | 0 ± 0 | 33% (n=1) | 0 ± 0 | 0 ± 0 | 0 ± 0 | 0 ± 0 | 0 ± 0 |
| *Rural* | 75 ± 28 | 0 ± 0 | 25 ± 28 | 0 ± 0 | 0 ± 0 | 0 ± 0 | 0 ± 0 | 0 ± 0 |
| **Tenrec** |  |  |  |  |  |  |  |  |
| *Urban* | 71 ± 6 | <1 ± <1 | 23 ± 4 | 17 ± 6 | <1 ± <1 | 9 ± 6 | 2 ± 2 | 1 ± 1 |
| *Rural* | 62 ± 18 | 0 ± 0 | 12 ± 9 | 12 ± 13 | 0 ± 0 | 7 ± 11 | 1 ± 2 | <1 ± <1 |
| **Wild Cat** |  |  |  |  |  |  |  |  |
| *Urban* | 74 ± 11 | 3 ± 3 | 11 ± 8 | 1 ± 2 | N.D. | N.D. | N.D. | N.D. |
| *Rural* | 77 ± 28 | 0 ± 0 | 20 ± 30 | 0 ± 0 | 0 ± 0 | 0 ± 0 | 0 ± 0 | 0 ± 0 |
| **Wild Pig** |  |  |  |  |  |  |  |  |
| *Urban* | 32 ± 20 | 0 ± 0 | 15 ± 8 | 62 ± 17 | 0 ± 0 | 29 ± 19 | 4 ± 3 | 11 ± 9 |
| *Rural* | 87 ± 16 | 0 ± 0 | 4 ± 5 | 23 ± 30 | 0 ± 0 | 19 ± 23 | 0 ± 0 | 0 ± 0 |

Averages ± 95% CI are shown and towns are replicates.
