# Supplementary material for: Capture, Movement, Trade, and Consumption of Mammals in Madagascar
Source: PLoS One. 2016 Feb 29;11(2):e0150305. doi: 10.1371/journal.pone.0150305 (PMC4771166; doi:10.1371/journal.pone.0150305)
Supplement: S7 Table — Includes individuals who had consumed wild meat at least once in a lifetime and the volume per year. Significant differences between towns are noted. Columns note whether towns or individual respondents are treated as replicates and depict the mean ± 95% Confidence Interval. Where individuals were used as replicates, sample sizes are listed behind each mean in parentheses. ND: No Data. (DOCX) [file pone.0150305.s015.docx]

**Table S7. The volume/rate of wild meat consumption, for individuals who had consumed wild meat at least once in a lifetime and the volume per year.**

| **Animal** | **Average number of times consumed in a lifetime**  ***(towns as replicates)*** | | **Average number of times consumed per year (pre-2009)**  ***(individuals as replicates)*** | | **Average number of times consumed per year (2009-2013)**  ***(individuals as replicates)*** | |
| --- | --- | --- | --- | --- | --- | --- |
|  | **Urban** | **Rural** | **Urban** | **Rural** | **Urban** | **Rural** |
| Bat | 53 ± 48* | 47 ± 47** | 38 ± 24 (51) | 3 ± 4 (5) | 14 ± 4 (162) | 7 ± 4 (32) |
| Civet | 12 ± 8* | 11 ± 6 | 19 ± 24 (12) | 1 ± <1 (6) | 9 ± 6 (19) | 3 ± 4 (16) |
| Fossa | 2 ± <1 | 4 ± 3 | 2 ± 2 (6) | <1 (1) | 1 ± 1 (5) | 2 ± 2 (8) |
| Lemur | 13 ± 7* | 30 ± 13 | 15 ± 10 (35) | 4 ± 2 (9) | 14 ± 16 (40) | 5 ± 2 (32) |
| Mongoose | 19 ± 18 | 9 ± 12* | 4 ± 3 (5) | 2 (1) | ND | 1 ± 2 (2) |
| Rats and mice | 3 ± 4 | 3 ± 0 | ND | | | |
| Tenrec | 49 ± 29* | 92 ± 77* | 17 ± 8 (51) | 2 ± <1 (3) | 13 ± 4 (185) | 9 ± 3 (90) |
| Wild Cat | 6 ± 4** | 17 ± 17 | 2 ± 2 (6) | ND | 1 ± <1 (8) | 1 ± <1 (9) |
| Wild Pig | 53 ± 24* | 135 ± 102 | 14 ± 8 (21) | ND | 18 ± 6 (111) | 8 ± 4 (29) |
| **All Wild Meat** | **86 ± 56*** | **117 ± 122*** | **6 ± 4***  **(towns as replicates)** | **<1 ± <1***  **(towns as replicates)** | **10 ± 6***  **(towns as replicates)** | **5 ± 5***  **(towns as replicates)** |
| Zebu (cattle) | ND  ND  ND | | | | 88 ± 20* | 67 ± 30* |
| Chicken |  |  |  |  | 30 ± 9* | 27 ± 12* |
| Pig |  |  |  |  | 54 ± 17 | 18 ± 8** |
| **All Domestic Meat** | **ND** | | | | **51 ± 11*** | **41 ± 17*** |

Significant differences between towns are noted. Columns note whether towns or individual respondents are treated as replicates and depict the mean ± 95% Confidence Interval. Where individuals were used as replicates, sample sizes are listed behind each mean in parentheses. ND: No Data.

* Significant differences between towns, Analysis of Variance, p < 0.05
** Marginal difference between towns, Analysis of Variance, 0.05 < p < 0.1
